# Cabo Verde’s Poaceae Flora: A Reservoir of Crop Wild Relatives Diversity for Crop Improvement

**DOI:** 10.3389/fpls.2021.630217

**Published:** 2021-02-01

**Authors:** Vanézia Rocha, Maria C. Duarte, Silvia Catarino, Ivani Duarte, Maria M. Romeiras

**Affiliations:** ^1^Linking Landscape, Environment, Agriculture and Food (LEAF), Instituto Superior de Agronomia (ISA), Universidade de Lisboa, Lisbon, Portugal; ^2^Centre for Ecology, Evolution and Environmental Changes (cE3c), Faculdade de Ciências, Universidade de Lisboa, Lisbon, Portugal; ^3^Forest Research Centre (CEF), Instituto Superior de Agronomia (ISA), Universidade de Lisboa, Lisbon, Portugal; ^4^Parque Natural do Monte Gordo, Ministério da Agricultura e Ambiente, Praia, Cabo Verde

**Keywords:** conservation strategies, crop wild relative (CWR), millets, plant genetic resources, grass flora, oceanic island, prioritization

## Abstract

Africa is home to important centers of origin and diversity of crop wild relatives (CWR), including many species adapted to adverse agroecological conditions, namely drought and poor soils. Plant genetic resources from Cabo Verde Islands have been poorly explored for their potential to supplement the genetic pool of cultivated species. In this paper we identify Cabo Verde’s CWR from the Poaceae family and provide a checklist of priority CWR *taxa*, highlighting those of particular conservation concern and the areas which should be the focus of the most intensive conservation efforts in these islands. Our results revealed that Cabo Verde archipelago is an important center of CWR diversity of West African crop millets, namely fonio (e.g., white fonio, *Digitaria exilis*, and black fonio, *Digitaria iburua*) and other African millets [e.g., pearl millet (*Cenchrus americanus* = *Pennisetum glaucum*), teff millet (*Eragrostis tef*), finger millet (*Eleusine coracana*), barnyard millet (*Echinochloa colona*), proso millet (*Panicum miliaceum*), and foxtail millet (*Setaria italica*)], which represent a diverse group of cereal crops, and important components in agriculture and food security of this country. Also, hotspot areas of diversity for *in situ* conservation were identified in Cabo Verde, as well as several populations occurring under extreme habitats conditions that are well adapted to drylands and poor soils. The evaluation of their potential for new ecologically important adaptive characteristics associated with tolerance to abiotic stresses is discussed. The survey of international Germplasm Banks revealed that very few accessions from Cabo Verde are conserved, contributing to the loss of genetic diversity of plant genetic resources in this archipelago. Particularly, the diversity of millets and the associated indigenous knowledge are critical for the food security and cultural identity of many poor farmers in Cabo Verde.

## Introduction

Crop Wild Relatives (CWR) are those species growing in natural habitats that are genetically related to food, fodder and forage crops, medicinal plants, condiments, ornamental and forestry species used by humankind (Maxted et al., 2006). CWR are valuable sources of adaptative traits, contributing to biotic and abiotic stress tolerance, and thereby allow crop improvements with a wide range of agronomical and nutritional benefits (Castañeda-Álvarez et al., 2016). As actual or potential gene donors, CWR have a wide range of important genetic traits due to their widespread adaptation to different habitats and to continuous *in situ* evolution and because they have not undergone domestication processes (Vincent et al., 2013; Zhang et al., 2017).

Considering an increasing population, the conservation of CWR diversity to ensure food security is of paramount importance and has been widely recognized by several world organizations, namely the Food and Agriculture Organization (Vincent et al., 2013). The Global Crop Diversity Trust has proposed projects like the “Global initiative to collect, conserve, and use crop wild relatives” (Dempewolf et al., 2014), and the 2030 agenda of Sustainable Development Goals (SDGs), implemented in 2015 by the United Nations, advocates the promotion of sustainable agriculture (SDGs2) by reinforcing resilience and adaptation to climate change (SDGs13) and preventing desertification and protection of biodiversity (domesticated and wild species) (SDGs15) (Sonesson et al., 2016). Like other Plant Genetic Resources, CWR are mainly threatened by the impacts of anthropogenic activities, such as habitat destruction, pollution and urbanization, and even competition by invasive species, but climate changes seem to be a particularly serious problem for this group (Ford-Lloyd et al., 2011; van Treuren et al., 2017; Teso et al., 2018; Allen et al., 2019). Some CWR may be passively conserved *in situ* due to strategies targeting other species, namely in protected areas, but specific conservation linked to CWR remains necessary.

The need for CWR conservation has been widely discussed (Heywood et al., 2007; Maxted et al., 2013) and the CWR inventory seems to be a crucial step to identify the conservation requirements (Zhang et al., 2017; Teso et al., 2018). Approaches to protect CWR diversity can involve an individual, national, regional or global level planning, with prioritization criteria that should ensure a successful conservation strategy with limited resources for implementation (Allen et al., 2019). The prioritization criteria can be adjusted according to the conservation strategy to be adopted, however, the main ones are: (i) economic value of the related crop, mainly its importance for human food and livestock supply; (ii) genetic potential as gene donor, priority being given to CWR that are more closely related to the crop; (iii) occurrence status of the CWR, as native, introduced or invasive on the geographical area in question; and iv) threat status (Maxted et al., 2013). Moreover, the Gene Pool (GP) (Harlan and de Wet, 1971) and Taxonomic Group (TG) (Maxted et al., 2006) concepts are used to determine the crossability between a crop and CWR. They are based on genetic and taxonomical relativeness, respectively GP and TG, and determine how closely a CWR relates to a crop and how easily they can cross (Maxted et al., 2006; Vincent et al., 2013).

The Poaceae family, with the third highest global priority among crop wild relatives, accounts for ca. 150 priority CWR distributed over 18 genera [*Aegilops* (=*Amblyopyrum*), *Agropyron*, *Avena*, *Cenchrus* (=*Pennisetum*), *Digitaria*, *Echinochloa*, *Eleusine, Elymus, Hordeum, Oryza, Panicum, Saccharum, Secale, Setaria, Sorghum, Tripsacum, Triticum*, and *Zea*] which, all except *Aegilops* and *Tripsacum*, belong to the global priority list of 92 CWR genera (Vincent et al., 2013). Among these genera are found most of the species that are wild relatives of the main consumption cereal crops (e.g., rice, wheat, maize, and oat), and other Poaceae food crops, such as sugarcane and sorghum, that substantially contribute to the human dietary energy (Vincent et al., 2013; Allen et al., 2019). Furthermore, in the semi-arid tropical regions of Africa and Asia, millets (small grain crops) are important sources of energy and protein for millions of persons living in developing countries (Amadou et al., 2013). The millets’ group comprises many different species, with pearl millet (*Cenchrus americanus* = *Pennisetum glaucum*), foxtail millet (*Setaria italica*), proso millet (*Panicum miliaceum*), and finger millet (*Eleusine coracana*) being the most important ones (Dwivedi et al., 2012; Amadou et al., 2013; Tadele, 2016). The maintenance of African millet diversity depends on agricultural, food and livelihood dynamics at the farmer level, since every community holds local cultivars to address their agroecological conditions, farming practices, and food needs (IPGRI, 2002). Small millets of the Poaceae family have been commonly mentioned as ‘smart foods’ or ‘nutri-cereals’ because they are more efficient in water and nitrogen use than major cereals like rice or maize (Muthamilarasan and Prasad, 2021). They grow in a diverse range of environmental conditions, as they are more tolerant to diseases, pests, and abiotic stresses (Vetriventhan et al., 2020).

To secure the long-term conservation, worldwide genebanks hold accessions of cultivated and wild germplasm of small millets, but only of the most important species, as is the case of finger millet, foxtail millet, and proso millet (Muthamilarasan and Prasad, 2021). Recently, Varshney et al. (2017) report the whole-genome sequence of pearl millet, providing an important resource to improve agronomic traits in arid environments and accelerate millets crop improvement.

Millets are well adapted to adverse climatic conditions (limited rainfall) and are mainly cultivated in marginal agricultural areas (Tadele, 2016), playing a major role in rural agriculture of West African countries, and particularly in the tropical dry islands of Cabo Verde (Teixeira and Barbosa, 1958). Although its agriculture is limited by a set of natural constraints (e.g., persistent drought periods, scarcity of quality soil, small territory available as farmland), this archipelago has shown considerable progresses toward overall development in the agriculture sector during the last two decades (Varela et al., 2020). Nevertheless, there is only official information on cultivation for two Poaceae species, *Zea mays* (maize) and *Saccharum officinarum* (sugarcane) (Monteiro et al., 2020). Maize is cultivated as a rainfed crop and used for human food and fodder; sugarcane is the most important irrigated crop and occupies the largest harvest area, its main purpose being the production of a by-product, the highly alcoholic drink “grog,” very much appreciated and exported as a national product (Monteiro et al., 2020).

Therefore, and despite the great importance of the Poaceae family as the dominant element of the native flora of Cabo Verde, the potential of plant genetic resources of these islands to supplement the genetic pool of cultivated species has been poorly explored. Considering the huge wealth of natural grass populations occurring in these islands under extreme environmental conditions, from dry lowlands to less dry altitude zones or even to subalpine regions (Neto et al., 2020), assessing the diversity of wild species with potential adaptive characteristics for abiotic stress tolerance would generate new data on varieties of Cabo Verde adapted to drought conditions (Essoh et al., 2020). This could contribute to valorise plant genetic resources and, in the future, to generate new eco-products with national economic impacts at the industry and economy levels.

Although this is one of the most important plant families in Cabo Verde and despite the importance of several crop species for humans and livestock, studies are scarce, particularly concerning the diversity of grasses in this archipelago. Moreover, there are threats, particularly to the endemic flora (Romeiras et al., 2016), requiring *in situ* (in natural habitats) and *ex situ* (in gene banks) conservation of the unique plant genetic resources that can assist the improvement of these crops and, consequently, ensure food security (Monteiro et al., 2020).

In this paper we intend to identify Cabo Verde’s CWR from the Poaceae family and to provide a checklist of priority CWR *taxa*, highlighting those of particular conservation concern. Based on a gap analysis, hotspot areas of Poaceae CWR diversity will be identified for this archipelago, in order to provide new data to propose future *in situ* conservation actions. Also, the total number of *ex situ* accessions will be identified for the African continent and, specifically, for Cabo Verde, in order to support future management of seed collection and conservation of local plant genetic resources. Finally, for the priority CWR Poaceae species identified, a comparative global analysis will be performed, and the results will be discussed in the context of West African food security.

## Materials and Methods

### Study Area

**FIGURE 1:**
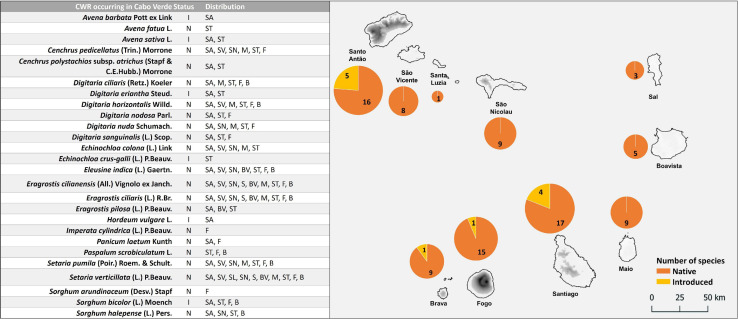
Crop wild relatives (CWR) Poaceae species occurring in Cabo Verde: status and distribution in the archipelago. The area of the circles is proportional to the number of species. Status: I, introduced; N, native. Island abbreviations: SA, Santo Antão; SV, São Vicente; SL, Santa Luzia; SN, São Nicolau; S, Sal; BV, Boavista; M, Maio; ST, Santiago; F, Fogo; B, Brava.

Cabo Verde is a North Atlantic Ocean archipelago and corresponds to the southernmost islands of Macaronesia. It is located at latitudes 14°45′ – 17°10′ N and longitudes 22°40′ – 25°20′ W, about 1,350 km south-west of the Canary Islands and ca. 560 km from Senegal’s coast. The archipelago (see Figure 1) has a total area of ca. 4,033 km^2^ and includes ten major islands distributed in three groups: Northern Islands [Santo Antão, São Vicente, Santa Luzia (the only uninhabited island), and São Nicolau]; Eastern Islands (Sal, Boavista and Maio); and the Southern Islands (Santiago, Fogo and Brava) (Duarte and Romeiras, 2009). Currently, the population is estimated at 556 thousand inhabitants but is expected to reach 679 thousand in 2050 (United Nations [UN], 2019).

Cabo Verde has a dry tropical climate with two well-marked seasons conditioning the distribution of its flora and vegetation (Neto et al., 2020). The long dry season of 8–10 months varies between November and July, and the sparse and irregular humid rainy season of 1–3 months, usually occurs from August to October (Monteiro et al., 2020). Mean annual temperature is usually around 25°C with low thermal amplitude, due to maritime influence; lowest monthly average temperatures normally occur in January or February (ca. 18°C), and the highest in September (ca. 27°C), however, temperatures as high as 35–40°C can occur in internal regions of the arid Eastern Islands (Duarte and Romeiras, 2009). The average annual relative air humidity ranges from 75% to more than 80% (Monteiro et al., 2020). Particularly, the topography of the islands contributes to significant spatial variations of flora and vegetation, with altitude and exposure to northeast trade winds leading to contrasting weather conditions (Duarte et al., 2008). The northern and southern islands are characterized by high mountains, offering a wide range of habitats over relatively short distances, whereas the eastern islands are lower and drier (Romeiras et al., 2015). Particularly, the lowland flora of Cabo Verde is markedly of Afrotropical origin and dominated by grass species, whereas the endemic mountain flora that mainly occurs on the north/northeast-facing slopes shows affinities with Madeiran and Canarian flora (Rivas-Martínez et al., 2017; Freitas et al., 2019).

### Poaceae Crop Wild Relatives’ Inventory and Data Collection

The Poaceae checklist from Cabo Verde was mainly based on data collected in previous fieldwork and inventories performed over the last two decades by M.C. Duarte, and on data from the literature (e.g., Lobin, 1986; Arechavaleta et al., 2005). Further data was obtained from specimens housed in worldwide herbaria (e.g., LISC, LISU, COI, K, and P), from Cabo Verde collections (e.g., in Parque Natural do Monte Gordo, São Nicolau Island), and information available in the Global Biodiversity Information Facility website (GBIF.org, 2020).

After completing the checklist of Cabo Verde CWR species, specific information was gathered on: (i) their status in Cabo Verde Islands - native or introduced; (ii) their distribution in the archipelago – number of islands where the species occur; (iii) their area of occupancy (AOO) calculated with a 1 km^2^ grid using occurrences of each species with valid coordinates, with QGIS v.3.10.5 software (QGIS Development Team, 2020); (iv) their world distribution; (v) their confirmed and potential traits and respective gene pool or taxon group, based on the Crop Wild Relative Project database (CWR, 2019); and (vi) their ethnobotany, according to information from GRIN – Germplasm Resources Information Network (USDA, 2020), Plant Resources of Tropical Africa (PROTA4U, 2020) and Cabo Verde herbarium data. Taxonomical nomenclature was checked according to Plants of the World Online – The Plant List (2013) and POWO (2019).

Crop species and their scientific names were gathered from the available literature (Dwivedi et al., 2012; Amadou et al., 2013; Vincent et al., 2013; Tadele, 2016). The native distribution of each crop species was obtained from GRIN (USDA, 2020), preferentially, and from POWO (2019). Additionally, for each crop species two measures of food supply – protein (g/100 g) and fat (g/100 g) – were retrieved from available literature (Kajuna, 2001; Belton and Taylor, 2004; Amadou et al., 2013; Chandi and Annor, 2016; Taylor, 2016; Williams et al., 2016; Wrigley, 2016) and the ICRISAT website (ICRISAT, 2020); also, two measures of global agricultural production – harvested area (ha) and production quantity (tons), using data from three recent years (2016–2018) (Food and Agricultural Organization of the United Nations [FAOSTAT], 2020) – were included. When the information on the agricultural production was aggregated for the listed crop, which is usually the case of millets, the values were disaggregated through division of the total value by all the identified crops.

### Importance and Priority Scores

For the Cabo Verde Poaceae wild relatives, the genetic potential as gene donor of each CWR species was identified, based on the gene pool concept (sublevel: GP1a, GP1b, GP2, and GP3) or, when this information was unavailable, on the taxon group concept (sublevel: TG1a, TG1b, TG2, TG3, TG4, and TG5). According to Maxted et al. (2006) and Vincent et al. (2013), the highest priority CWR is the one that can most easily cross with the crop, namely GP1b, GP2, when using gene pool concept or TG1b, TG2, and TG3, in the case of the taxon group concept. The genetic potential as gene donor is one of the most used parameters to establish conservation priorities.

The importance of each associated crop was estimated by applying the “Importance Score” (IS), adapted from Castañeda-Álvarez et al. (2016). The Importance Score was produced: initially, by dividing the food supply (protein and fat) and the agricultural production (harvested area and production quantity) by the maximum existing value across all crops; then, the food supply and agricultural production metrics were averaged separately; finally, the Importance Score was produced by averaging the mean food supply and mean agricultural production values (Castañeda-Álvarez et al., 2016).

In this study, a “Priority Score” (PS) for each CWR was calculated based on nine criteria (see Table 1), two directly related with the associated crops (I and II) and seven with the crop wild relatives themselves (III to IX). The prioritization criteria were adapted from Heywood et al. (2007) and Maxted et al. (2013). For each class, within each criterion, a value of 1–3 was assigned concerning conservation importance (1 – low, 2 – medium, 3 – high) as follows: (I) crop Importance Score – 1 (IS ≤ 0.2), 2 (0.2 < IS ≤ 0.4), 3 (IS > 0.4); (II) native distribution of the crop – 1 (native out Africa), 2 (native to Africa, excluding West Africa), 3 (native to West Africa and other regions); (III) genetic potential as gene donor of the CWR – 1 (GP3 or TG3), 2 (GP2 or TG2), 3 (GP1 or TG1); (IV) number of associated crops – 1, 2, or 3 (respectively, with one, two or three crops); (V) status in Cabo Verde – 1 (introduced, native from regions out of Africa), 2 (introduced, native in Africa but not in Cabo Verde), 3 (native in Cabo Verde); (VI) distribution in Cabo Verde – 1 (≥6 islands), 2 (4–5 islands), 3 (1 – 3 islands); (VII) area of occupancy, as a proxy to the threat status in Cabo Verde – 1 (>40 km^2^), 2 (21 – 40 km^2^), 3 (≤20 km^2^); (VIII) world distribution – 1 (native out Africa), 2 (native to Africa, excluding West Africa), 3 (native to West Africa, and other regions); (IX) ethnobotanical uses – 1 (≤2 uses), 2 (3 – 4 uses), 3 (>4 uses). For each CWR species, PS results from the sum of all the values assigned to it and priority classes were established as follows: low - up to 18; medium - up to 21; high - higher than 21.

**TABLE 1:** Summary of the nine criteria and the scores assigned to each CWR and its associated crop.

	**No.**	**Criteria**	**Score 1 - Low**	**Score 2 - Medium**	**Score 3 - High**
Associated crop	I	Crop Importance Score (IS)	IS ≤ 0.2	0.2 < IS ≤ 0.4	IS > 0.4
	II	World native distribution of the crop	Native out Africa	Native to Africa, excluding West Africa	Native to West Africa and other regions
CWR	III	CWR genetic potential as gene donor	GP3 or TG3	GP2 or TG2	GP1 or TG1)
	IV	Number of associated crops	1	2	3
	IV	CWR status in Cabo Verde	Introduced, native from regions out of Africa	Introduced, native in Africa but not in Cabo Verde	Native in Cabo Verde
	VI	Distribution in Cabo Verde (number of islands)	≥6 islands	4–5 islands	1–3 islands
	VII	Area of occupancy (AOO)	>40 km^2^	21–40 km^2^	≤20 km^2^
	VIII	World native distribution	Native out Africa	Native to Africa, excluding West Africa	Native to West Africa and other regions
	IX	Ethnobotanical uses	≤2 uses	3–4 uses	>4 uses

For the CWR species related to more than one crop (as, for instance, *Echinochloa colona* to barnyard millet and Indian barnyard millet, see Table 2), the Priority Score (see Table 3) was calculated considering, for each criterion, the highest assigned score (e.g., if a CWR belongs to GP1 of one crop and to GP3 of another, a score of 3 was given). For comparative analyzes of crops, the mean PS was gathered adding the individual values of all the CWR associated to a given crop and dividing the total by the corresponding CWR number (Castañeda-Álvarez et al., 2016).

**TABLE 2:** List of **(A)** Poaceae crops considered in this study, their Importance Score and native distributions; and **(B)** the associated crop wild relatives occurring in Cabo Verde Islands and information on their gene pool, native status, and uses.

**(A) Crops**				**(B) Associated crop wild relatives**			
**Crop name**	**Scientific name**	**Importance Score**	**Native distribution**	**CWR occurring in Cabo Verde**	**Gene pool**	**Status in Cabo Verde**	**Uses**
Barley	*Hordeum vulgare* L.	0.504	Africa (N); Europe; Asia	Hordeum vulgare*	GP1	I	H, F, Fo, E, M, Me
Barnyard millet	*Echinochloa colona* (L.) Link, *Echinochloa crus-galli* (L.) P.Beauv.	0.304	Africa; Middle East; South Asia. Europe (E)	Echinochloa colona* Echinochloa crus-galli*	GP1 GP1	N I	H, F^CV^, Fo, E H, F, E, Me
Finger millet	*Eleusine coracana* subsp. *coracana* (L.) Gaertn.	0.189	Africa; Asia (Temperate)	*Eleusine indica*	GP1	N	H, F^CV^, Fo, M, Me
Fonio	*Digitaria exilis* (Kippist) Stapf (white fonio) & *Digitaria iburua* Stapf (black fonio)	0.304	Africa (W)	*Digitaria ciliaris*	GP3	N	H, F^CV^, E
				*Digitaria eriantha*	GP3	I	F, Fo, E, M, Me
				*Digitaria horizontalis*	GP3	N	H, F
				*Digitaria nodosa*	GP3	N	F^CV^
				*Digitaria nuda*	GP3	N	H, F
				*Digitaria sanguinalis*	GP3	N	F, Fo, E
Foxtail millet	*Setaria italica* (L.) P.Beauv.	0.383	Africa (N); Europe; Asia	*Setaria verticillata*	GP2	N	H, F^CV^, Fo, E, M, Me
				*Setaria pumila*	GP3	N	H, F^CV^, E, M, Me
Indian barnyard millet	*Echinochloa frumentacea* Link.	0.203	Asia (S)	*Echinochloa colona*	GP1	N	H, F^CV^, Fo, E
				*Echinochloa crus-galli*	GP3	I	H, F, E, Me
Japanese barnyard millet	*Echinochloa esculenta* (A.Braun) H.Scholz	0.203	Asia (E)	*Echinochloa crus-galli*	GP1	I	H, F, E, Me
Kodo millet	*Paspalum scrobiculatum* L.	0.210	Africa; Pacific; Asia; Australia	Paspalum scrobiculatum*	GP1	N	H, F, Fo, E, M, Me
Oat	*Avena sativa* L.	0.537	Middle East	Avena sativa*	GP1	I	H, F, Fo, Me
				*Avena fatua*	GP1	N	F, Fo, Me
				*Avena barbata*	GP3	I	F
Pearl millet	*Cenchrus americanus* (L.) Morrone	0.449	Africa (W, C, S)	*Cenchrus pedicellatus*	GP3	N	F, Fo, M, Me
				*Cenchrus polystachios* subsp. *atrichus*	GP3	N	H, F, E, M, Me
Proso millet	*Panicum miliaceum* L.	0.272	Asia (Temperate)	*Panicum laetum*	GP3	N	H, F, Me
Sorghum	*Sorghum bicolor* (L.) Moench	0.469	Africa	Sorghum bicolor*	GP1	I	H, F, Fo, E, M, Me
				*Sorghum arundinaceum*	GP1	N	H, F^CV^, M, Me, O
				*Sorghum halepense*	GP2	N	H, F, Me
Sugarcane	*Saccharum officinarum* L.	0.502	Oceania (New Guinea)	*Imperata cylindrica*	GP3	N	H, F, E, M, Me, O
				*Sorghum bicolor*	GP3	I	H, F, Fo, E, M, Me
Teff (millet)	*Eragrostis tef* (Zuccagni) Trotter	0.254	Africa (E); Middle East	*Eragrostis pilosa*	GP1	N	H, F, Me
				*Eragrostis cilianensis*	TG2^a^	N	H, F^CV^, Fo, E, M
				*Eragrostis ciliaris*	TG2^a^	N	H, F^CV^, M, Me

CWR taxon: * The wild form of the crop. Status: I, introduced; N, native. CWR uses: H, human consumption; F, forage; Fo, fodder; E, environmental; M, materials; Me, medicinal; O, other; ^*CV*^, with information reported for Cabo Verde. (a) according to Ingram and Doyle (2007).

**TABLE 3:** Poaceae CWR occurring in Cabo Verde, their native status, distribution, area of occupancy, gene pool, scoring of the prioritization criteria and conservation priority levels.

**CWR taxon**	**Native status**	**Number of islands**	**Area of occupancy (km^2^)**	**Gene pool (higher level)**	**Criteria and respective scores^a^**									**Priority Score**	**Priority category**
					**Associated crop**		**CWR**								
					**I**	**II**	**III**	**IV**	**V**	**VI**	**VII**	**VIII**	**IX**		
*Avena barbata*	I	1	1	GP3	3	1	1	1	1	3	3	2	1	16	Low
*Avena fatua*	N	1	< 0.5	GP1	3	1	3	1	3	3	3	2	2	21	Medium
*Avena sativa*	I	2	3	GP1	3	1	3	1	1	3	3	1	2	18	Low
*Cenchrus pedicellatus*	N	6	23	GP3	3	3	1	1	3	1	2	3	2	19	Medium
*Cenchrus polystachios* subsp. *atrichus*	N	2	3	GP3	3	3	1	1	3	3	3	3	3	23	High
*Digitaria ciliaris*	N	5	50	GP3	2	3	1	1	3	2	1	3	2	18	Low
*Digitaria eriantha*	I	2	1	GP3	2	3	1	1	2	3	3	2	3	20	Medium
*Digitaria horizontalis*	N	6	21	GP3	2	3	1	1	3	1	2	1	1	15	Low
*Digitaria nodosa*	N	3	11	GP3	2	3	1	1	3	3	3	3	1	20	Medium
*Digitaria nuda*	N	5	56	GP3	2	3	1	1	3	2	1	3	1	17	Low
*Digitaria sanguinalis*	N	3	2	GP3	2	3	1	1	3	3	3	2	2	20	Medium
*Echinochloa colona*	N	5	35	GP1	2	1	3	2	3	2	2	3	2	20	Medium
*Echinochloa crus-galli*	I	1	< 0.5	GP1	2	1	3	3	1	3	3	1	2	19	Medium
*Eleusine indica*	N	7	54	GP1	1	3	3	1	3	1	1	3	3	19	Medium
*Eragrostis cilianensis*	N	9	45	TG2	2	2	2	1	3	1	1	3	3	18	Low
*Eragrostis ciliaris*	N	9	38	TG2	2	2	2	1	3	1	2	3	2	18	Low
*Eragrostis pilosa*	N	3	< 0.5	GP1	2	2	3	1	3	3	3	3	2	22	High
*Hordeum vulgare*	I	1	2	GP1	3	2	3	1	1	3	3	2	3	21	Medium
*Imperata cylindrica*	N	1	3	GP3	3	1	1	1	3	3	3	3	3	21	Medium
*Panicum laetum*	N	2	1	GP3	2	1	1	1	3	3	3	3	2	19	Medium
*Paspalum scrobiculatum*	N	3	14	GP1	2	3	3	1	3	3	3	3	3	24	High
*Setaria pumila*	N	7	40	GP3	2	2	1	1	3	1	2	3	3	18	Low
*Setaria verticillata*	N	10	75	GP2	2	2	2	1	3	1	1	2	3	17	Low
*Sorghum arundinaceum*	N	1	1	GP1	3	3	3	1	3	3	3	3	3	25	High
*Sorghum bicolor*	I	4	7	GP1	3	3	3	2	2	2	3	3	3	24	High
*Sorghum halepense*	N	4	20	GP2	3	3	2	1	3	2	3	2	2	21	Medium

**^*a*^Criteria: (I) crop importance; (II) world native distribution of the crop; (III) CWR genetic potential as gene donor; (IV) number of associated crops; (V) CWR status in Cabo Verde; (VI) distribution in Cabo Verde (number of islands); (VII) area of occupancy; (VIII) world native distribution; (IX) ethnobotanical uses*.*

### Diversity Hotspots and *in situ* Conservation Gap Analysis

The distribution of Poaceae CWR in Cabo Verde was estimated with occurrence data collected from previous fieldwork, studied specimens housed in worldwide herbaria and information available at the GBIF website (GBIF.org, 2020). Specimens without geographical coordinates were georeferenced following the Guide to Best Practices for Georeferencing (Chapman and Wieczorek, 2006) and using Google Earth Pro 7.3.2.5491 (Serea, 2018). Duplicate records, i.e., with the same collector and the same number of collection, were excluded. The final dataset with 675 occurrences with geographical coordinates was used in the analysis.

The altitudinal distribution of each CWR species was estimated based on the interception of the occurrence records with the map of altitude provided by CGIAR-CSI Consortium for Spatial Information (CGIAR-CSI Consortium for Spatial Information, 2020) at a resolution of 90 m. To summarize these data, we built a boxplot graph, using R version 3.6.0. (R Development Core Team, 2020).

Based on georeferenced occurrence data, we also constructed a species richness map using QGIS v.3.10.5 (QGIS Development Team, 2020). This map presents the number of species occurring in each cell of 4 km^2^, allowing to identify the areas of greatest diversity of CWR species in Cabo Verde. Species occurrences and diversity hotspots were then overlaid with the national network of protected areas of Cabo Verde, downloaded from Infra-estrutura de Dados Espaciais de Cabo Verde (IDE-CV, 2020). This analysis aimed to assess the coverage and efficiency of the network of protected areas to preserve CWR species, identifying the main conservation gaps.

### *Ex situ* Conservation Analysis

The status of *ex situ* conservation of Cabo Verde’s Poaceae CWR in worldwide genebanks was assessed through the Genesys Database (Genesys, 2020). Comprehensive data for West Africa and specifically for Cabo Verde were identified in order to support future management of seed collection and conservation of local plant genetic resources.

## Results

### Diversity of Cabo Verde Poaceae CWR

The inventory of the Poaceae family revealed that ca. 123 native and introduced *taxa* occur in Cabo Verde Islands. Twenty-six species are CWR, including five native species (*Eleusine indica, Eragrostis cilianensis, Eragrostis ciliaris, Setaria pumila*, and *Setaria verticillata*) that are widespread in the archipelago, occurring in more than 7 islands, and seven species (*Avena barbata*, *Avena sativa*, *Digitaria eriantha, Hordeum vulgare, Imperata cylindrica, Panicum laetum*, and *Sorghum arundinaceum*) that occur only in a single island (Figure 1). Santiago has the highest diversity of species (21), including 17 native and four introduced ones (Figure 1). Santo Antão, also with 21 CWR, is the island hosting more introduced species (*A. barbata, A. sativa, D. eriantha, H. vulgare* and *Sorghum bicolor*). The lowest number of CWR was found in Sal (3 species), and Santa Luzia (1 species – *S. verticillata*).

Among the CWR species, six are the wild forms of the identified crop (i.e., *A. sativa*, *E. colona*, *Echinochloa crus-galli*, *H. vulgare*, *Paspalum scrobiculatum*, and *S. bicolor*) and three (*E. colona*, *E. crus-galli*, and *S. bicolor*) are associated with more than one crop. Of the CWR occurring in Cabo Verde, twenty are native to the islands and the remaining six *taxa* are introduced; four of the latter have a native distribution range in Africa (Table 2, more details in Supplementary Table 1). Three crops (fonio, pearl millet, and sorghum) have native distributions exclusively in the African continent, and six (barley, barnyard millet, finger millet, foxtail millet, kodo millet, and teff) have a native range that includes Africa; the other five crops (Indian barnyard millet, Japanese barnyard millet, oat, proso millet, and sugarcane) are non-native to Africa (Table 2).

**FIGURE 2:**
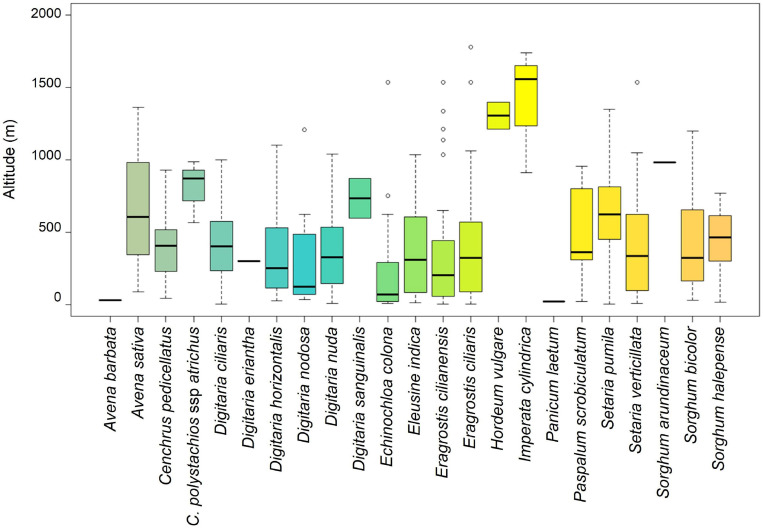
Altitudinal distribution of CWR records in Cabo Verde Islands. The boxplots show the minimum (at the bottom of the chart, at the end of the vertical line), first quartile, Q1, (the bottom edge of the box), the median (line in the center of the box), third quartile, Q3 (the top edge of the box), the maximum (at the top end of the vertical line), and outliers (circles). Species represented with a single line have only one recorded occurrence. Species without records (*Avena fatua, Echinochloa crus-galli* and *Eragrostis pilosa*) were not included in this figure.

Based on 675 occurrence records (Supplementary Table 2), we analyzed the altitudinal distribution of the studied CWR species; it ranges from sea level (*D. ciliaris, E. cilianensis*, and *S. pumila*) to 1,780 m (*E. ciliaris*) (Figure 2). Most species have a large altitudinal distribution, occurring from low altitude to more than 1,000 m. The species *Digitaria nodosa*, *E. colona*, and *E. cilianensis* were identified mainly in lower areas, with median altitudes of 320, 201, and 313 m, respectively. The species *H. vulgare* and *I. cylindrica* stand out for their ability to grow at high altitude, with medians of 1,306 and 1,560 m, respectively.

### Crop Importance and CWR Priority Scores

Among the Poaceae crops considered in this study, five can be considered of high importance according to the Importance Score (Table 2): pearl millet, sorghum, sugarcane, barley, and oat. Eight crops are of medium importance: Indian barnyard millet, Japanese barnyard millet, kodo millet, teff (millet), proso millet, barnyard millet, fonio, and foxtail millet. Only the finger millet was classified as of low crop importance.

**FIGURE 3:**
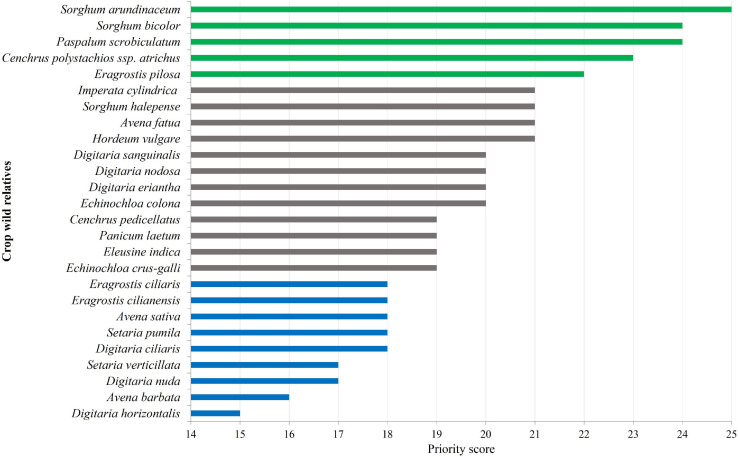
Priority for collecting and conserving the CWR Poaceae. Different colors represent different priority categories: green – highest (PS > 21), gray – medium (21 ≥ PS > 18), and blue – low (PS ≤ 18).

The “Priority Scores” (PS) for crop wild relatives, ranging from 15 to 25, with score 15 corresponding to low priority CWR (*Digitaria horizontalis*), and CWR with the highest priority (score 25) being *S. arundinaceum* (Table 3, Figure 3, and Supplementary Table 3). From the 26 identified CWR, five (19.2%) *taxa* were ranked as high priority, 12 (46.2%) as medium priority and nine (34.6%) as low priority (Figure 3). In terms of gene pool, 10 *taxa* were classified as GP1 (38.5%), four as GP2 and TG2 (15.4%), and 12 as GP3 (46.1%) (Table 3). Three species are wild relative to more than one crop: *E. crus-galli*, GP1 of barnyard millet and Japanese barnyard millet, and GP3 of Indian barnyard millet*; E. colona*, GP1 of barnyard millet and Indian barnyard millet; and *S. bicolor*, GP1 of sorghum and GP3 of sugarcane.

**FIGURE 4:**
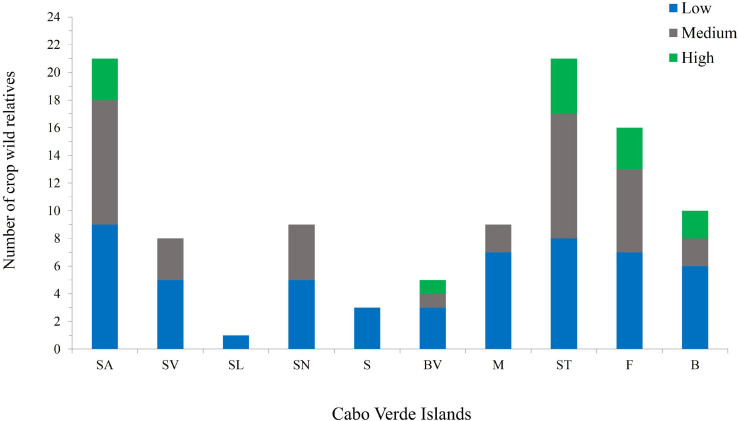
Number of priority CWR Poaceae species per island and their priority categories; total CWR for Cabo Verde archipelago is 26. Different colors represent different priority categories: green – highest; gray – medium; blue – low. Island abbreviations: SA, Santo Antão; SV, São Vicente; SL, Santa Luzia; SN, São Nicolau; S, Sal; BV, Boavista; M, Maio; ST, Santiago; F, Fogo; B, Brava.

Except for Boavista, mountain islands (maximum altitude between 900 and 2,800 m) are those with high-priority *taxa* (Figure 4 and Supplementary Table 4) such as *S. arundinaceum*, *S. bicolor, P. scrobiculatum, Cenchrus polystachios* subsp. *atrichum* and *Eragrostis pilosa*. In Santa Luzia only one CWR occurs (*S. verticillata*); this may be related to the fact that this island has been subject of less botanical exploration over the last decades than the inhabited islands, as well as to fewer available habitats due to its limited area.

**FIGURE 5:**
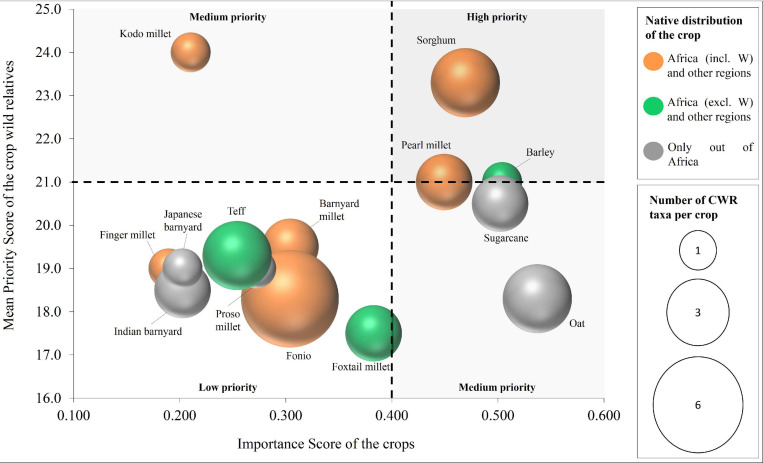
Comparison of the importance of the 14 Poaceae crops studied and their CWR in Cabo Verde. The Importance Score concerns the food supply and agricultural production metrics of the crops and the mean Priority Score represents the nine criteria used as a proxy to prioritize the CWR (for details see section “Importance and Priority Scores”). The size of the circles indicates the number of CWR taxa per crop. The colours indicate the native distribution of the crop.

A comparative view of the importance of Poaceae crops and their associated CWR in Cabo Verde is represented in Figure 5 (more details in Supplementary Table 5). Among the nine crops with native distribution in the African continent, six occur in West Africa, with kodo millet, sorghum and pearl millet being those with more associated high-priority CWR [*P. scrobiculatum* (kodo millet); *S. arundinaceum* and *S. bicolor* (sorghum); *C. polystachios* subsp. *atrichus* (pearl millet)]. Among priority species to collect and conserve, the CWR of sorghum and pearl millet should be highlighted (upper right part of Figure 5: PS > 21; IS > 0.4). Fonio is the crop with more associated CWR in Cabo Verde (6), with a native distribution exclusive to West Africa and all the associated CWR being used as forage (Table 2); however, the low Importance Score of the crop together with the fact that all the fonio CWR present in Cabo Verde belong to GP3, place this group as of low priority. Although this crop presents a medium Importance Score (0.304) it is an important African crop, which supports animal livestock and some human supply. Oat, sugarcane and barley, non-native to Cabo Verde, are among the most important crops; their associated CWR in Cabo Verde are mainly introduced species, with native distributions in Africa.

The CWR species themselves have important uses. We identified seven species with six different uses, six species with five uses, and eight species with four uses; nine species have three or less uses (Table 2). The most common use is as fodder (26 *taxa*), followed by human consumption (20 *taxa*) and medicinal applications (18 *taxa*).

Information regarding confirmed and potential traits are only available for barley, oat and sorghum crops, and mainly concerning abiotic, agronomic and biotic traits, such as drought tolerance, yield improvement and pathogen resistance, respectively. For the remaining crops, and especially millets, no information is available.

### *In situ* Conservation: Hotspots and Conservation Gap Analysis

**FIGURE 6:**
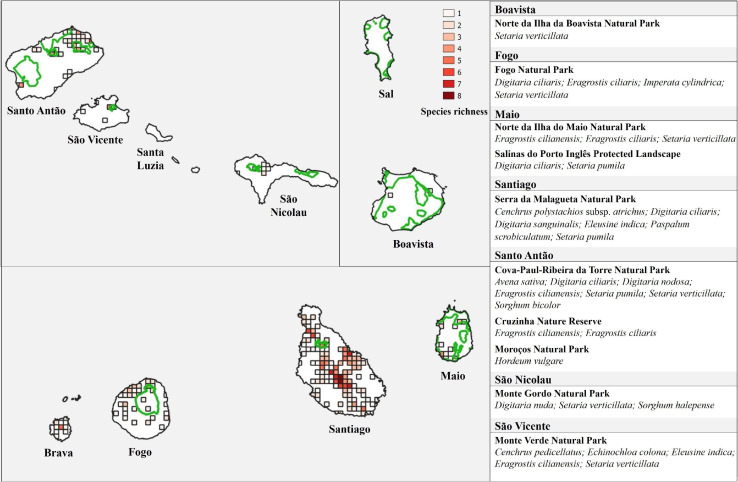
Species richness of the CWR Poaceae species in Cabo Verde Islands, in 2 km × 2 km cells, and network of protected areas (green lines). The CWR *taxa* occurring in each protected area are listed (right).

The main diversity hotspots are found in Santiago and Santo Antão (Figure 6). The maximum of eight species per cell of 2 km × 2 km was found in Santiago, between Pico da Antónia and Rui Vaz, including one high priority (*P. scrobiculatum*) and two medium priority species (*Cenchrus pedicellatus* and *E. indica*). Another important hotspot was found in the same island, near Ponta de Santa Cruz, with seven species, including one of high conservation priority (*P. scrobiculatum*) and four of medium priority (*C. pedicellatus, E. colona, E. indica*, and *S. halepense*). In Santo Antão, the highest diversity was found in Paúl (northeast of the island), with six species, two of them of medium conservation priority (*D. nodosa* and *E. colona*).

The most diverse areas in terms of species richness are generally outside the protected areas (Figure 6). Only 8.1% of the occurrence records, corresponding to 18 species (three species of high priority, eight of medium priority, and seven of low priority), are found within protected areas. Cova-Paúl-Ribeira da Torre Natural Park, in Santo Antão, hosts the highest number of CWR with seven species, however, only one of them is of high conservation priority and one is of medium priority (*S. bicolor* and *D. nodosa*, respectively). Six taxa, including two of high priority (*C. polystachios* subsp. *atrichus* and *P. scrobiculatum*) and two of medium priority (*D. sanguinalis* and *E. indica*), are found in Serra da Malagueta Natural Park. Five CWR species occur in Monte Verde Natural Park, three of them are medium priority (*C. pedicellatus*, *E. colona*, and *E. indica*). Eight species – *A. barbata, A. fatua, D. eriantha, D. horizontalis, E. crus-galli, E. pilosa* (high priority), *P. laetum*, and *S. arundinaceum* (high priority) –, were not found in protected areas.

### *Ex situ* Conservation

The analyses of the accessions present in global genebanks reveal that 25 species (96.1%) of CWR mentioned in the present work are currently conserved *ex situ* (Table 4). Considering the accessions collected worldwide, *H. vulgare* (252,545 accessions), a medium priority species, is the CWR with the highest number of accessions, followed by the high priority species *S. bicolor* (123,473 accessions), and the low priority species *Avena sativa* (53,617 accessions). Three taxa (11.5%) have 1,000 – 2,000 accessions, seven taxa (26.9%) have 100 – 1,000 accessions, and 12 taxa (46.2%) have 1 – 100 accessions. The only species without accession is *Digitaria nuda*.

**TABLE 4:** Accessions of the studied species available in international Germplasm Banks.

**CWR occurring in Cabo Verde**	**Provenance and number of accessions**			**Countries holding accessions**	**Total accessions**
	**West Africa**	**Africa (except West Africa)**	**Other Regions**		
*Avena barbata*	0	240 [Morocco (199); Libya (41)]	988	>10	1228
*Avena fatua*	0	0	1876	>10	1876
Avena sativa*	0	0	53617	>10	53617
*Cenchrus pedicellatus*	30 [Niger (17); Mali (9); Nigeria (2); Burkina Faso (1); Mauritania (1)]	62 [Cameroon (56); Central African Republic (3); Ethiopia (3)]	61	5	153
*Cenchrus polystachios* subsp. *atrichus*	2 [Burkina Faso (1); Mali (1)]	1 [Central African Republic (1)]	0	1	3
*Digitaria ciliaris*	1 [Niger (1)]	8 [Tanzania (3); Mozambique (2); Kenya (1); Madagascar (1); Republic of South Africa (1)]	9	5	18
*Digitaria eriantha*	0	584	113	6	697
*Digitaria horizontalis*	6 [Burkina Faso (5); Nigeria (1)]	0	1	4	7
*Digitaria nodosa*	0	4 [Kenya (4)]	1	2	5
*Digitaria nuda*	0	0	0	0	0
*Digitaria sanguinalis*	0	2 [Malawi (2)]	32	>10	34
Echinochloa colona*	19 [Mali (19)]	21[Kenya (8); Republic of South Africa (4); Botswana (3); Ethiopia (2); Malawi (2); Sudan (2)]	520	7	660
Echinochloa crus-galli*	0	0	350	10	350
*Eleusine indica*	10 [Nigeria (7); Ghana (3)]	76 [Uganda (48); Kenya (22); Democratic Republic of Congo (4); Burundi (2)]	82	>10	168
*Eragrostis cilianensis*	9 [Mali (6); Burkina Faso (3)]	13 [Kenya (8); Madagascar (4); Ethiopia (1)]	12	6	34
*Eragrostis ciliaris*	3 [Mali (2); Burkina Faso (1)]	9[Kenya (8); Madagascar (1)]	4	2	16
*Eragrostis pilosa*	9 [Burkina Faso (6); Mali (3)]	23 [Kenya (18); Ethiopia (3); Madagascar (2)]	19	6	51
Hordeum vulgare*	0	18391 [Ethiopia (all)]	234154	>10	252545
*Imperata cylindrica*	0	9 [Madagascar (3); Kenya (2); Tanzania (2); Malawi (1); Republic of South Africa (1)]	12	4	21
*Panicum laetum*	28 [Mali (24); Niger (2); Mauritania (1); Burkina Faso (1)]	0	0	2	28
Paspalum scrobiculatum*	13 [Mali (9); Niger (4)]	79 [Kenya (29); Zimbabwe (17); Tanzania (7); Uganda (7); Madagascar (5); Republic of South Africa (5); Ethiopia (9)]	1022	7	1114
*Setaria pumila*	0	6 [Cameroon (4); Kenya (2)]	81	10	87
*Setaria verticillata*	1 [Burkina Faso (1)]	4 [Botswana (4)]	28	9	33
*Sorghum arundinaceum*	11 [Mali (11)]	311 [Sudan (145); Kenya (42); Republic of South Africa (34); Uganda (30); Angola (19); Ethiopia (18); Egypt (13); Chad (10)]	108	9	430
Sorghum bicolor*	6729 [Nigeria (3488); Mali (3240); Cabo Verde (1; Accession number – IS 27941; PGRFA doi: 10.18730/NS7TT)]	37265 [Ethiopia (12112); Sudan (11444); Kenya (6526); Zimbabwe (4152); Uganda (3031)]	79479	>10	123473
*Sorghum halepense*	0	17 [Sudan (9); Angola (4); Republic of South Africa (4)]	186	> 10	203

CWR taxon: * = The wild form of the crop. Due to the floristic affinities with Cabo Verde and West African floras, the provenance of the accessions is provided. Accession number and DOI are included to the only Cabo Verde accession.

Most high priority species are generally well represented in world genebanks: *S. arundinaceum* has a total of 430 accessions, but only 11 were collected in West Africa, namely in Mali; *S. bicolor* has more than 120,000 accessions; *P. scrobiculatum* has 1,114 accessions, 13 of them collected in West Africa; *C. polystachios* subsp. *atrichus* is very poorly represented, with only 3 accessions, two of them from West Africa; and *E. pilosa* has 51 accessions, including nine from West Africa.

Considering only the accessions collected in Cabo Verde, we found one, of *S. bicolor*, hosted in the International Crop Research Institute for the Semi-arid Tropics (ICRISAT) in India.

## Discussion

Climate change in Sub-Saharan Africa (SSA) has been impacting water resources, and agricultural and food systems, particularly during the first decade of the 21st century, the warmest decade on record (Rickards and Howden, 2012; Hartmann et al., 2013). Cabo Verde Islands are highly susceptible to climate change due to consecutive years of drought and their poor soil structure, intensified by scarce vegetation cover (Neto et al., 2020).

In drylands, the importance of Poaceae species extends from the cultivation of grasslands and erosion control to, and especially, uses by humans (e.g., supply of cereals) and livestock (e.g., fodder and forage) (Capstaff and Miller, 2018; Mganga et al., 2019). More than half of the population’s food supply is provided by three grass crops (i.e., rice, wheat, and maize) which are particularly important in developing countries where they provide food security and nutrition to local populations (Tadele, 2016).

Our study identified 26 Cabo Verde’s CWR from the Poaceae family. This archipelago is an important center of wild diversity of African crop millets, namely of fonio (e.g., white fonio *D. exilis*, and black fonio, *D. iburua*) and other African millets, such as: pearl millet (*C. americanus*), teff millet (*E. tef*), finger millet (*E. coracana*), barnyard millet (*E. colona*), proso millet (*P. miliaceum*), and foxtail millet (*S. italica*). African millets represent a diverse group of cereal crops, which are well adapted to adverse agroecological conditions (Garí, 2002). Millets and their wild forms represent critical plant genetic resources for the agriculture and food security of poor farmers who inhabit arid, infertile, and marginal lands (Teixeira et al., 2013). The Food and Agriculture Organization has announced the year 2023 as ‘International Year of Millets’, recognizing the potential of these crops to fight malnutrition and hunger in developing countries (Muthamilarasan and Prasad, 2021).

Both millets and sorghum CWR of Cabo Verde occur under extreme climatic conditions in this archipelago, being presumably more resilient to climate change. Some of the CWR species found in Cabo Verde, namely *S. bicolor, H. vulgare*, and *A. fatua*, have already shown high tolerance to droughts and saline environments (Dinari et al., 2013; Ogbaga et al., 2014; Gous et al., 2015). Moreover, a study conducted by Gurney et al. (2002) revealed that *Sorghum arundinaceum* is highly tolerant to infection by *Striga hermonthica* and *Striga asiatica* root hemiparasites known to attack crops and cause great losses in production. Also, *Sorghum halepense* is comparatively less susceptible to downy mildew infection (caused by *Peronosclerospora sorghi*) than the associated crop (Kamala et al., 2002). Thus, Cabo Verde’s CWR could be a valuable source of resistance genes to increase the tolerance of their related crops to biotic and abiotic stresses.

According to Adhikari et al. (2015), the estimated yield loss by the end of this century due to climate change is less than 20% for African millets and sorghum, whereas for other grain crops, such as wheat, a reduction of as much as 72% is foreseen, and for rice and soybean, of up to 45%.

In Cabo Verde, the greatest richnesses of CWR were found in Santiago, Santo Antão, and Fogo islands, with ten or more different taxa; the species richness roughly increased with the area and maximum altitude of the island, which is related with more available habitats, in agreement with other studies on endemic flora (Romeiras et al., 2015, 2016). Also, these islands have more agricultural activity but, except for the cultivation of sugarcane, which is the primary irrigated crop of Cabo Verde (Monteiro et al., 2020), there are no official reports on the cultivation/production of the Poaceae crops included in this study. Some CWR are the wild forms of cultivated crops (e.g., *E. colona, E. crus-galli, H. vulgare, P. scrobiculatum*, and *S. bicolor*) and most of them grow on marginal agricultural areas, mainly of maize, and on grazing areas. Barnyard millet (*E. colona*) occurs in lowland areas of Santo Antão, São Vicente, São Nicolau, Maio and Santiago; it is well adapted to arid ecosystems and tolerates soils with poor fertility. Nevertheless, *H. vulgare* and *I. cylindrica*, the CWR of sugarcane (*S. officinarum*) stand out for their ability to develop at high altitudes (averaging 1,306 and 1,560 m, respectively). Currently, sugarcane and maize are the most cultivated grass crops in Cabo Verde, but other species are used in their wild forms, for human consumption and, particularly, as forage (Monteiro et al., 2020). The cultivated wild forms of millets could offer a more sustainable food source than their related major crops because they are more efficient in the use of water and nitrogen (Muthamilarasan and Prasad, 2021).

Among the Poaceae species ranked as of high priority for further collection in Cabo Verde, there are *C. polystachios* subsp. *atrichus*, *P. scrobiculatum, S. arundinaceum, S. bicolor*, and CWRs associated with fonio; all of these species are used in the archipelago for human and animal consumption, as well as for medicinal purposes (Romeiras et al., 2011).

In the past, these food plants have played an important role in the diet and traditional medicine of African communities (Catarino et al., 2016; Havik et al., 2018; Akinola et al., 2020). Several of these species are important African crops (Amadou et al., 2013; Tadele, 2016), but in Cabo Verde there are no evidences of their cultivation, most of them being used for livestock grazing (Barbosa, 1961) or has building materials (e.g., thatching with *I. cylindrica*).

Some of the reported Poaceae species are used since the colonization of Cabo Verde (Barbosa, 1961; Romeiras et al., 2011). Since the mid-1500s, Cabo Verde has become the subject of numerous travel descriptions reporting on the local flora and on the introduction of new crops such as sugarcane, maize and cotton (Romeiras et al., 2018). Other reports, by the chronicler Valentim Fernandes, confirmed the large production and abundance of sorghum and pearl millet in the West African region, from where they were imported at very early stages of the colonization of this archipelago (Romeiras et al., 2014). These African millets, together with rice, were the most important crops during the settlement of Cabo Verde and the basis of the population’s diet (Santos and Torrão, 1998). In the 17th century, pearl millet (*milho zaburro*, *milho de maçaroca* or *milho branco*, as was locally called), began to be intensively cultivated and made this archipelago self-sufficient in cereals, only needing to import cereals from West Africa in drought years (Teixeira da Mota and Carreira, 1966). The introduction and cultivation of maize (*Zea mays* L.) gradually replaced the role that African millets had until the 18th century (Torrão, 1995). Ancient reports refer that maize was known by the Portuguese since the 16th century, due to regular trade between Cabo Verde and the Antilles, where this species is native. However, the cultivation of African millets, more adapted to poor soils and with less water requirements, was more appropriate than that of the American maize (Santos and Torrão, 1998). Since the end of the journeys to the Antilles and the establishment of regular trade with Brazil, maize became the food basis of the Cabo Verdean population (Santos and Torrão, 1998) and is currently a dominant part of their diet (Monteiro et al., 2020). The importance of historical factors and the role played by Cabo Verde in the Atlantic navigation during the 16th–19th centuries (Romeiras et al., 2020), contributed to change and determine the present composition of Cabo Verde’s flora, with more than 70% of exotic species, most of them introduced for food purposes.

Therefore, the valorisation of the plant genetic resources related to millet crops and their wild relatives is of major importance to fight hunger and ensure food and nutrition security in Cabo Verde. This archipelago is still very dependent on food importations, particularly in years of prolonged and severe droughts, as happened in 2017/2018 (Monteiro et al., 2020). Adding to the adverse natural conditions, it is a small and fragmented insular country, with inherent difficulties in connections with West Africa and Europe, as well as between islands, posing problems to the rapid provision of food (either locally produced or imported) to more inaccessible rural areas, namely in Brava, São Nicolau, and Maio Islands. Despite recent progress in reducing extreme poverty, ca. 30% of Cabo Verde’s population still lives in multidimensional poverty with poor health care, lack of education, and inadequate living standards, which is a strong economic obstacle to meet the food needs of a large section of the population (Varela et al., 2020).

Notwithstanding the recognized importance of African millets, information available on online databases, such as GRIN (USDA, 2020), revealed that data on these species and their wild relatives are still limited. Also, the worldwide CWR inventory^1^ revealed that there is no information about confirmed traits for millets. Therefore, *ex situ* conservation of plant genetic resources of Cabo Verde must be a national priority in response to the rapid loss of agricultural biodiversity, as there is only one accession (*S. bicolor*, Genesys, 2020) available for Cabo Verdean Poaceae CWRs in genebanks. New expeditions must be performed in these islands, to collect CWR species growing in threatened habitats, and targeted to priority species with few accessions stored in worldwide genebanks, such as *C. polystachios* subsp. *atrichus, E. pilosa*, *I. cylindrica, P. laetum*, and *S. arundinaceum*. Most of these taxa have native distribution ranges in West Africa and are important for human consumption and to feed livestock. Moreover, and although their use is less widespread, some species of the genus *Urochloa* (=*Brachiaria*) present in Cabo Verde are considered small millets in Africa. Such is the case of *Urochloa deflexa* (Schumach.) H.Scholz [=*Brachiaria deflexa* (Schumach.) C.E.Hubb. ex Robyns] (guinea millet), used as food in the Sudan-Zambezi and Yemenite regions in its wild form and, occasionally, cultivated in the highlands of the Fouta Djalon (Portères, 1976), and of *Urochloa ramosa* (L.) T.Q.Nguyen [=*Brachiaria ramosa* (L.) Stapf] (browntop millet) more widespread as forage, but also used as food in India (Kimata et al., 2000). In Cabo Verde, these species are mostly referred to as forage. There is no record of genetic material from Cabo Verde, where one of the five existing *Urochloa* species is endemic [=*Urochloa caboverdiana* (Conert & C.Kohler) Veldkamp, Potdar & S.R.Yadav], reinforcing the need for its *ex situ* conservation.

Although *ex situ* conservation has had more worldwide success than *in situ* conservation, probably because of its facility of access by users and lower cost (De-Zhu and Pritchard, 2009; Díez et al., 2018), the establishment of the Protected Areas Network in Cabo Verde has already contributed to safeguard the archipelago’s natural heritage and endemic species (MAAP, 2004; Romeiras et al., 2016). Our study was able to identify hotspot areas for *in situ* conservation of CWR populations across the archipelago, and species were identified and correlated with habitat conditions, namely to detect which ones are better adapted to drylands, highlands, and poor soils in these islands.

## Final Remarks

The benefits of grass crops, namely African millets, and their ancestral use in Cabo Verde, were highlighted in our study, which also alerts to the need of rescuing cultural values, and to the consumer’s unawareness of the advantages of these plants, well adapted to the very dry conditions of this archipelago. However, the adverse natural environmental conditions of a small and fragmented insular country such as Cabo Verde, with inherent difficulties in inter-island transportation, hinder the supply of food products to rural populations. So, it is necessary to produce more and with better quality under various limitations, such as marginal lands, water shortage, soil degradation, or climate change (Castañeda-Álvarez et al., 2016). In this context, viable approaches to improve food security are crucial, and the systematic use of CWR in crop improvement appears essential to face the increasing pressure on food production while maintaining natural diversity. Cabo Verde’s plant diversity faces increasing threats (Romeiras et al., 2016) due to desertification processes, and native species remain a viable sustainable land management option to fight degradation in these tropical dry islands.

## Data Availability Statement

The original contributions generated for this study are included in the article/Supplementary Material, further inquiries can be directed to the corresponding author.

## Author Contributions

MD and MR: conceptualization and supervision. VR, MD, SC, and MR: methodology and writing—review and editing. VR and SC: formal analysis. MD, VR, and ID: field surveys. VR: investigation. MR: writing—original draft preparation. All authors have read and agreed to the published version of the manuscript.

## Conflict of Interest

The authors declare that the research was conducted in the absence of any commercial or financial relationships that could be construed as a potential conflict of interest.

## Footnotes

1 https://www.cwrdiversity.org/checklist/

## References

[B1] Adhikari U. Nejadhashemi A. P. Woznicki S. A. (2015). Climate change and eastern Africa: a review of impact on major crops. *Food Energy Sec.* 4 110–132. 10.1002/fes3.61

[B2] Akinola R. Pereira L. M. Mabhaudhi T. de Bruin F. M. Rusch L. (2020). A review of indigenous food crops in africa and the implications for more sustainable and healthy food systems. *Sustainability* 12:3493 10.3390/su12083493 PMC7116648 33520291

[B3] Allen E. Gaisberger H. Magos Brehm J. Maxted N. Thormann I. Lupupa T. (2019). A crop wild relative inventory for Southern Africa: a first step in linking conservation and use of valuable wild populations for enhancing food security. *Plant Gen. Resourc.* 17 1–12. 10.1017/S1479262118000515

[B4] Amadou I. Gounga M. E. Le G.-W. (2013). Millets: nutritional composition, some health benefits and processing – a review. *Food Sci. Nutr.* 25 501–508. 10.9755/ejfa.v25i7.12045

[B5] Arechavaleta M. Zurita N. Marrero M. C. Martín J. L. (eds) (2005). *Lista Preliminar de Espécies Silvestres de Cabo Verde (Hongos, Plantas y Animales Terrestres).* Santa Cruz de Tenerife: Consejería de Medio Ambiente y Ordenación Territorial, 155.

[B6] Barbosa L. A. G. (1961). Subsídios para um dicionário utilitário e glossário dos nomes vernáculos das plantas de arquipélago de Cabo Verde. *Garcia Orta Sér. Bot.* 9 37–91.

[B7] Belton P. S. Taylor J. R. N. (2004). Sorghum and millets: protein sources for Africa. *Trends Food Sci. Technol.* 15 94–98. 10.1016/j.tifs.2003.09.002

[B8] Capstaff N. M. Miller A. J. (2018). Improving the yield and nutritional quality of forage crops. *Front. Plant Sci.* 9:535. 10.3389/fpls.2018.00535 29740468 PMC5928394

[B9] Castañeda-Álvarez N. P. Khoury C. K. Achicanoy H. A. Bernau V. Dempewolf H. Eastwood R. J. (2016). Global conservation priorities for crop wild relatives. *Nat. Plants* 2:16022. 10.1038/nplants.2016.22 27249561

[B10] Catarino L. Havik P. Romeiras M. M. (2016). Medicinal plants of Guinea-Bissau: therapeutic applications, ethnic diversity and knowledge transfer. *J. Ethnopharmacol.* 183 71–94. 10.1016/j.jep.2016.02.032 26923540

[B11] CGIAR-CSI Consortium for Spatial Information (2020). *SRTM 90m Digital Elevation Database v4.1.* Available online at: https://cgiarcsi.community/data/srtm-90m-digital-elevation-database-v4-1/ (accessed on 16 June 2020).

[B12] Chandi G. K. Annor G. A. (2016). *Millet Minor: Overview. In: Encyclopedia of Food Grains.* Amsterdam: Elsevier Inc., 199–208.

[B13] Chapman A. D. Wieczorek J. (2006). *Guide to Best Practices for Georeferencing.* Copenhagen: Global Biodiversity Information Facility.

[B14] CWR (2019). *The Crop Wild Relative Project.* Available online at: https://www.cwrdiversity.org/checklist/ (accessed on 15 March 2020)

[B15] Dempewolf H. Eastwood R. J. Guarino L. Khoury C. K. Müller J. V. Toll J. (2014). Adapting agriculture to climate change: a global initiative to collect, conserve, and use crop wild relatives. *Agroecol. Sustain. Food Syst.* 38 369–377. 10.1080/21683565.2013.870629

[B16] De-Zhu L. Pritchard H. W. (2009). The science and economics of ex situ plant conservation. *Trends Plant Sci.* 14 614–621. 10.1016/j.tplants.2009.09.005 19818672

[B17] Díez M. J. De la Rosa L. Martín I. Guasch L. Cartea M. E. Mallor C. (2018). Plant genebanks: present situation and proposals for their improvement. The case of the Spanish Network. *Front. Plant Sci.* 9:1794. 10.3389/fpls.2018.01794 30564263 PMC6288731

[B18] Dinari A. Meighani F. Farzami S. M. (2013). Effects of salinity and drought stress on germination and seedling growth of *Avena fatua* L. and *Phalaris minor* L. *Iran. J. Plant Physiol.* 3 665–671.

[B19] Duarte M. C. Rego F. Romeiras M. M. Moreira I. (2008). Plant species richness in the Cape Verde Islands – eco-geographical determinants. *Biodivers. Conserv.* 17 453–466. 10.1007/s10531-007-9226-y

[B20] Duarte M. C. Romeiras M. M. (2009). “Cape Verde Islands,” in *Encyclopedia of Islands*, eds Gillespie R. Clague D. (Berkeley: University of California Press), 143–148. 10.1525/9780520943728-033

[B21] Dwivedi S. Upadhyaya H. Senthilvel S. Hash C. Fukunaga K. Diao X. (2012). Millets: genetic and genomic resources. *Plant Breed. Rev.* 35 247–375. 10.1002/9781118100509.ch5

[B22] Essoh A. P. Monteiro F. Pena A. R. Pais M. S. Moura M. Romeiras M. M. (2020). Exploring glucosinolates diversity in Brassicaceae: a genomic and chemical assessment for deciphering abiotic stress tolerance. *Plant Physiol. Biochem.* 150 151–161. 10.1016/j.plaphy.2020.02.032 32142988

[B23] Food and Agricultural Organization of the United Nations [FAOSTAT] (2020). *Food and Agricultural Data.* Available online at: http://www.fao.org/faostat/en/#home (accessed on 27 April 2020)

[B24] Ford-Lloyd B. V. Schmidt M. Armstrong S. J. Barazani O. Engels J. Hadas R. (2011). Crop wild relatives – undervaluated, underutilized and under threat? *BioScience* 61 559–565. 10.1525/bio.2011.61.7.10 33021500

[B25] Freitas R. Romeiras M. Silva L. Cordeiro R. Madeira P. González J. A. (2019). Restructuring of the ‘Macaronesia? Biogeographic unit: a marine multitaxon biogeographical approach. *Sci. Rep.* 9:15792. 10.1038/s41598-019-51786-6 31690834 PMC6831653

[B26] Garí J. A. (2002). “Review of the African millet diversity,” in *Proceedings of the International Workshop on Fonio, Food Security and Livelihood Among the Rural Poor in West Africa*, (Bamako: IPGRI/IFAD), 19–22.

[B27] GBIF.org (2020). *GBIF Occurrence Download.* Available online at: 10.15468/dl.9gm3g3 (accessed on 30 June 2020).

[B28] Genesys (2020). *Plant Genetic Resources Accession.* Available online at: https://www.genesys-pgr.org/ (accessed 19 July 2020)

[B29] Gous P. W. Gilbert R. G. Fox G. P. (2015). Drought-proofing barley (*Hordeum vulgare*) and its impact on grain quality: a review. *J. Inst. Brew.* 121 19–27. 10.1002/jib.187

[B30] Gurney A. L. Press M. C. Scholes J. D. (2002). Can wild relatives of sorghum provide new sources of resistance or tolerance against Striga species? *Weed Res.* 42 317–324. 10.1046/j.1365-3180.2002.00291.x

[B31] Harlan J. R. de Wet J. M. J. (1971). Towards a rational classification of cultivated plants. *Taxon* 20 509–517. 10.2307/1218252

[B32] Hartmann D. L. Klein Tank A. M. G. Rusticucci M. Alexander L. V. Brönnimann S. Charabi Y., et al. (eds) (2013). “Observations: atmosphere and surface,” in *Climate Change 2013: The Physical Science Basis. Contribution of Working Group I to the Fifth Assessment Report of the Intergovernmental Panel on Climate Change*, eds Stocker T. F. Qin D. Plattner G.-K. Tignor M. Allen S. K. Boschung J. (Cambridge: Cambridge University Press).

[B33] Havik P. J. Monteiro F. Catarino S. Correia A. M. Catarino L. Romeiras M. M. (2018). Agro-economic transitions in guinea-bissau (West Africa): historical trends and current insights. *Sustainability* 10:3408 10.3390/su10103408

[B34] Heywood V. Casas A. Ford-Lloyd B. Kell S. Maxted N. (2007). Conservation and sustainable use of crop wild relatives. *Agric. Ecosyst. Environ.* 121 245–255. 10.1016/j.agee.2006.12.014

[B35] ICRISAT (2020). *ICRISAT.* Available online at: https://www.icrisat.org/ (accessed on 9 April 2020)

[B36] IDE-CV (2020). *Infra-Estrutura de Dados Espaciais de Cabo Verde.* Available online at: https://idecv-ingt.opendata.arcgis.com/ (accessed on 06 October 2020).

[B37] Ingram A. L. Doyle J. J. (2007). *Eragrostis* (Poaceae): monophyly and infrageneric classification. *Aliso J. Syst. Evol. Bot.* 23 595–604. 10.5642/aliso.20072301.44

[B38] IPGRI (2002). *Programme for Neglected and Underutilised Species.* Rome: International Plant Genetic Resources Institute.

[B39] Kajuna S. T. A. R. (2001). “Millet: post-harvested operations,” in *FAO INPhO – Post-Harvest Compendium*, eds Mejía D. Lewis B. (Morogoro: Sokone University of Agriculture).

[B40] Kamala V. Singh S. D. Bramel P. J. Rao D. M. (2002). Sources of resistance to downy mildew in wild and weedy sorghums. *Crop Sci.* 42 1357–1360. 10.2135/cropsci2002.1357

[B41] Kimata M. Ashok E. G. Seetharam A. (2000). Domestication, cultivation and utilization of two small millets, *Brachiaria ramosa* and *Setaria glauca*, Poaceae in South India. *Econ. Bot.* 54 217–227. 10.1515/9783110806373.409

[B42] Lobin W. (1986). Katalog der von den Kapverdischen Inseln beschriebenen Taxa höherer Pflanzen (Pteridophyta & Phanerogamae). *Cour. Forschungsinst. Senckenberg* 81 93–164.

[B43] MAAP (2004). *Livro Branco sobre o Estado do Ambiente em Cabo Verde.* North Melbourne VIC: MAAP.

[B44] Maxted N. Ford-Lloyd B. V. Jury S. Kell S. Scholten M. (2006). Towards a definition of a crop wild relative. *Biodivers. Conserv.* 15 2673–2685. 10.1007/s10531-005-5409-6

[B45] Maxted N. Magos Brehm J. Kell S. (2013). *Resource Book for Preparation of National Conservation Plans for Crop Wild Relatives and Landraces.* Birmingham: University of Birmingham.

[B46] Mganga K. Z. Nyariki D. M. Musimba N. K. Mwang’ombe A. W. (2019). “Indigenous grasses for rehabilitating degraded African drylands,” in *Agriculture and Ecosystem Resilience in Sub Saharan Africa*, eds Y. Bamutaze, S. Kyamanywa, B. R. Singh, G. Nabanoga, and R. Lal (Cham: Springer), 53–68.

[B47] Monteiro F. Fortes A. Ferreira V. Essoh A. P. Gomes I. Correia A. M. (2020). Current status and trends in Cabo Verde agriculture. *Agronomy* 10:74 10.3390/agronomy10010074

[B48] Muthamilarasan M. Prasad M. (2021). Small millets for enduring food security amidst pandemics. *Trends Plant Sci.* 26 33–40. 10.1016/j.tplants.2020.08.008 32900620 PMC7474701

[B49] Neto C. Costa J. C. Figueiredo A. Capelo J. Gomes I. Vitória S. (2020). The role of climate and topography in shaping the diversity of plant communities in Cabo Verde Islands. *Diversity* 12:80 10.3390/d12020080

[B50] Ogbaga C. C. Stepien P. Johnson G. N. (2014). Sorghum (*Sorghum bicolor*) varieties adopt strongly contrasting strategies in response to drought. *Physiol. Plant.* 152 389–401. 10.1111/ppl.12196 24666264

[B51] Portères R. (1976). “African Cereals: *Eleusine*, Fonio, Black Fonio, Tejf, *Brachiaria*, *Paspalum*, *Pennisetum*, and African Rice,” in *Origins of African Plant Domestication*, ed. Harlan J. R. (Mouton: The Hague), 409–452.

[B52] POWO (2019). *Plants of the World Online. Facilitated by the Royal Botanic Gardens, Kew.* Available online at: http://www.plantsoftheworldonline.org/ (accessed on 15 January 2020)

[B53] PROTA4U (2020). *Plant Resources of Tropical Africa.* Available online at: https://www.prota4u.org/database/ (accessed on 25 March 2020)

[B54] QGIS Development Team (2020). *QGIS Geographic Information System. Open Source Geospatial Foundation Project.* Available online at: http://qgis.osgeo.org (accessed October 6, 2020).

[B55] R Development Core Team (2020). *R: A Language and Environment for Statistical Computing.* Vienna: R Foundation for Statistical Computing.

[B56] Rickards L. Howden S. M. (2012). Transformational adaptation: agriculture and climate change. *Crop Past. Sci.* 63 240–250.

[B57] Rivas-Martínez S. Lousã M. Costa J. C. Duarte M. C. (2017). Geobotanical survey of the Cabo Verde Islands (West Africa). *Int. J. Geobot. Res.* 7 1–103.

[B58] Romeiras M. M. Carine M. Duarte M. C. Catarino S. Dias F. S. Borda-de-Água L. (2020). Bayesian methods to analyze historical collections in time and space: a case study using Cabo Verde Endemic Flora. *Front. Plant Sci.* 11:278 10.3389/fpls.2020.00278 PMC7083154 32231676

[B59] Romeiras M. M. Catarino L. Torrão M. M. Duarte M. C. (2011). Diversity and origin of medicinal exotic flora in Cape Verde Islands. *Plant Ecol. Evol.* 142 214–225. 10.5091/plecevo.2011.560

[B60] Romeiras M. M. Catarino S. Filipe A. Magalhães M. Duarte M. C. Beja P. (2016). Species conservation assessments in small islands: the consequences of precautionary versus evidentiary attitudes. *Conserv. Lett.* 9 275–280. 10.1111/conl.12212

[B61] Romeiras M. M. Catarino S. Gomes I. Fernandes C. Costa J. C. Caujapé-Castells J. (2015). IUCN red list assessment of the Cape Verde endemic flora: towards a Global strategy for plant conservation in Macaronesia. *Bot. J. Linnean Soc.* 180 413–425. 10.1111/boj.12370

[B62] Romeiras M. M. Duarte M. Francisco-Ortega J. Catarino L. Havik P. (2018). Recovering plant data for guinea-bissau: implications for biodiversity knowledge of West Africa. *Diversity* 10:109 10.3390/d10040109

[B63] Romeiras M. M. Duarte M. C. Santos-Guerra A. Carine M. Francisco-Ortega J. (2014). Botanical exploration of the Cape Verde Islands: from the pre-Linnaean records and collections to late 18th century floristic accounts and expeditions. *Taxon* 63 625–640. 10.12705/633.37

[B64] Santos M. E. M. Torrão M. M. (1998). “Entre l’Amérique et L’Afrique, les iles du Cap-Vert et São Tomé: Les cheminements des *milhos* (mil, sorgho et maïs),” in *Plantes et paysages d’Afrique: une histoire à explorer*, ed. Chastanet M. (Paris: Karthala Editions).

[B65] Serea R. (2018). *Google Earth Pro 7, 3.2.5491.* Available online at: https://www.neowin.net/news/google-earth-pro-7325491/ (accessed on 06 March 2020)

[B66] Sonesson C. Davidson G. Sachs L. (2016). *Mapping Mining to the Sustainable Development Goals: A Preliminary Atlas.* Cologny: World Economic Forum.

[B67] Tadele Z. (2016). “Drought adaptation in millets. Chapter 26,” in *Abiotic and Biotic Stress in Plants - Recent Advances and Future Perspectives*, eds Shanker A. K. Shanker C. (Rijeka: IntechOpen).

[B68] Taylor J. R. N. (2016). Millet pearl: overview. *Encycl. Food Grains* 1, 190–198. 10.1016/B978-0-12-394437-5.00011-5

[B69] Teixeira A. J. S. Barbosa L. A. G. (1958). “A agricultura do arquipélago de Cabo Verde: Cartas agrícolas, problemas agrários,” in *Memórias da Junta de Investigações do Ultramar*, 2 178.

[B70] Teixeira, da Mota A. Carreira A. (1966). “Milho Zaburro” and “Milho Maçaroca” in Guinea and in the Islands of Cabo Verde. *J. Int. Afric. Inst.* 36 73–84.

[B71] Teixeira E. I. Fischer G. van Velthuizen H. Walter C. (2013). Global hot-spots of heat stress on agricultural crops due to climate change. *Agric. For. Meteorol.* 170 206–215.

[B72] Teso M. L. R. Lamas E. T. Parra-Quijano M. de la Rosa L. Fajardo J. Iriondo J. M. (2018). National inventory and prioritization of crop wild relatives in Spain. *Genet. Resourc. Crop. Evol.* 65 1237–1253. 10.1007/s10722-018-0610-0

[B73] The Plant List (2013). *Version 1.1.* Available online at: www.theplantlist.org/ (accessed on 25 September 2019)

[B74] Torrão M. M. (1995). *Dietas Alimentares. Transferências e Adaptações nas Ilhas de Cabo Verde (1460–1540).* Lisboa: Instituto de Investigação Científica Tropical, 108.

[B75] United Nations [UN] (2019). *Department of Economic and Social Affairs, Population Division. World Population Prospects 2019.* Available online at: http://esa.un.org/unpd/wpp/index.htm (accessed on 2 May 2020)

[B76] USDA (2020). “Agricultural research service, and national plant germplasm system,” in *Germplasm Resources Information Network (GRIN-Taxon*omy). Beltsville, MD: National Germplasm Resources Laboratory.

[B77] van Treuren R. Hoekstra R. van Hintum T. J. L. (2017). Inventory and prioritization for the conservation of crop wild relatives in The Netherlands under climate change. *Biol. Conserv.* 216 123–139. 10.1016/j.biocon.2017.10.003

[B78] Varela D. Monteiro F. Vidigal P. Silva L. Romeiras M. M. (2020). Mechanisms implemented for the sustainable development of agriculture: an overview of Cabo Verde performance. *Sustainability* 12:5855 10.3390/su12145855

[B79] Varshney R. Shi C. Thudi M. (2017). Pearl millet genome sequence provides a resource to improve agronomic traits in arid environments. *Nat. Biotechnol.* 35 969–976. 10.1038/nbt.3943 28922347 PMC6871012

[B80] Vetriventhan M. Azevedo V. C. R. Upadhyaya H. D. (2020). Genetic and genomic resources, and breeding for accelerating improvement of small millets: current status and future interventions. *Nucleus* 63 217–239. 10.1007/s13237-020-00322-3

[B81] Vincent H. Wiersema J. Kell S. Fielder H. Dobbie S. Castañeda-Álvarez N. P. (2013). A prioritized crop wild relative inventory to help underpin global food security. *Biol. Conserv.* 167 265–275. 10.1016/j.biocon.2013.08.011

[B82] Williams I. O. Onyenweaku E. O. Atangwho I. J. (2016). Nutritional and antimicrobial evaluation of *Saccharum officinarum* consumed in Calabar. Nigeria. *Afric. J. Biotechnol.* 15 1789–1795. 10.5897/AJB2015.14877

[B83] Wrigley C. W. (2016). An overview of the family of cereal grains prominent in the world agriculture. *Encycl. Food Grains* 1 73–85. 10.1016/B978-0-12-394437-5.00006-1

[B84] Zhang H. Mittal N. Leamy L. J. Barazani O. Song B. H. (2017). Back into the wild – apply untapped genetic diversity of wild relatives for crop improvement. *Evol. Appl.* 10 5–24. 10.1111/eva.12434 28035232 PMC5192947

