# Supplementary material for: Cabo Verde’s Poaceae Flora: A Reservoir of Crop Wild Relatives Diversity for Crop Improvement
Source: Front Plant Sci. 2021 Feb 1;12:630217. doi: 10.3389/fpls.2021.630217 (PMC7901987; doi:10.3389/fpls.2021.630217)
Supplement: Supplementary file 1 [file Data_Sheet_1.docx]

Supplementary Material

**Supplementary Table 1.** Species of Poaceae Crop Wild Relatives occurring in Cabo Verde with details on tribe, subfamily, native status, life cycle, photosynthetic pathways, and islands of occurrence.

| **Taxa** | **Tribe** | **Subfamily** | **Status in Cabo Verde** | **Life cycle** | **Photosynthetic pathways** | **Islands ^a^** |
| --- | --- | --- | --- | --- | --- | --- |
| *Avena barbata* Pott ex Link | Poeae | Pooideae | Introduced | Annual | C3 | SA |
| *Avena fatua* L. | Poeae | Pooideae | Native | Annual | C3 | ST |
| *Avena sativa* L. | Poeae | Pooideae | Introduced | Annual | C3 | SA,ST |
| *Cenchrus pedicellatus* (Trin.) Morrone | Paniceae | Panicoideae | Native | Annual | C4 | SA, SV, SN, M, ST, F |
| *Cenchrus polystachios* subsp. *atrichus* (Stapf & C.E.Hubb.) Morrone | Paniceae | Panicoideae | Native | Perennial | C4 | SA, ST |
| *Digitaria ciliaris* (Retz.) Koeler | Paniceae | Panicoideae | Native | Annual | C4 | SA, M, ST, F, B |
| *Digitaria eriantha* Steud. | Paniceae | Panicoideae | Introduced | Perennial | C4 | SA,ST |
| *Digitaria horizontalis* Willd. | Paniceae | Panicoideae | Native | Annual | C4 | SA, SV, M, ST, F, B |
| *Digitaria nodosa* Parl. | Paniceae | Panicoideae | Native | Perennial | C4 | SA, ST, F |
| *Digitaria nuda* Schumach. | Paniceae | Panicoideae | Native | Annual | C4 | SA, SN, M, ST, F |
| *Digitaria sanguinalis* (L.) Scop. | Paniceae | Panicoideae | Native | Annual | C4 | SA, ST, F |
| *Echinochloa colona* (L.) Link | Paniceae | Panicoideae | Native | Annual | C4 | SA, SV, SN, M, ST |
| *Echinochloa crus-galli* (L.) P.Beauv. | Paniceae | Panicoideae | Introduced | Annual | C4 | ST |
| *Eleusine indica* (L.) Gaertn. | Cynodonteae | Chloridoideae | Native | Annual | C4 | SA, SV, SN, BV, ST,F, B |
| *Eragrostis cilianensis* (All.) Vignolo ex Janch. | Eragrostideae | Chloridoideae | Native | Annual | C4 | SA, SV, SN, S, BV, M, ST, F,B |
| *Eragrostis ciliaris* (L.) R.Br. | Eragrostideae | Chloridoideae | Native | Annual | C4 | SA, SV, SN, S, BV, M, ST, F, B |
| *Eragrostis pilosa* (L.) P.Beauv. | Eragrostideae | Chloridoideae | Native | Annual | C4 | SA, BV, ST |
| *Hordeum vulgare* L. | Triticeae | Pooideae | Introduced | Annual | C3 | SA |
| *Imperata cylindrica* (L.) P.Beauv. | Andropogoneae | Panicoideae | Native | Perennial | C4 | F |
| *Panicum laetum* Kunth | Paniceae | Panicoideae | Native | Annual | C4 | SA, F |
| *Paspalum scrobiculatum* L. | Paspaleae | Panicoideae | Native | Perennial | C4 | ST, F, B |
| *Setaria pumila* (Poir.) Roem. & Schult. | Paniceae | Panicoideae | Native | Annual | C4 | SA, SV, SN, M, ST, F, B |
| *Setaria verticillata* (L.) P.Beauv. | Paniceae | Panicoideae | Native | Annual | C4 | SA, SV, SL, SN, S, BV, M, ST, F, B |
| *Sorghum arundinaceum* (Desv.) Stapf | Andropogoneae | Panicoideae | Native | Perennial | C4 | F |
| *Sorghum bicolor* (L.) Moench | Andropogoneae | Panicoideae | Introduced | Annual | C4 | SA, ST,F, B |
| *Sorghum halepense* (L.) Pers. | Andropogoneae | Panicoideae | Native | Perennial | C4 | SA, SN, ST, B |

**^a^** Island abbreviations: SA=Santo Antão; SV=São Vicente; SL=Santa Luzia; SN=São Nicolau; S=Sal; BV=Boavista; M=Maio; ST=Santiago; F=Fogo; B=Brava

**Supplementary Table 2.** Occurrence records of Poaceae crop wild relatives in Cabo Verde.

| **Specimen** | **Year of record** | **Island** | **Latitude** | **Longitude** | **Altitude (m)** |
| --- | --- | --- | --- | --- | --- |
| *Avena barbata* | 1864 | Santo Antão | 17.176706 | -25.065502 | 28 |
| *Avena sativa* | 1993 | Santo Antão | 17.108767 | -25.072127 | 1365 |
| *Avena sativa* | 1956 | Santo Antão | 17.157848 | -25.080781 | 603 |
| *Avena sativa* | 1956 | Santo Antão | 17.117968 | -24.991783 | 85 |
| *Cenchrus pedicellatus* | 1991 | Fogo | 14.976396 | -24.464845 | 360 |
| *Cenchrus pedicellatus* | 1980 | Santiago | 15.110133 | -23.666881 | 401 |
| *Cenchrus pedicellatus* | 1980 | Santiago | 15.107217 | -23.670293 | 525 |
| *Cenchrus pedicellatus* | 1934 | São Vicente | 16.869618 | -24.933916 | 630 |
| *Cenchrus pedicellatus* | 1934 | São Vicente | 16.869441 | -24.933719 | 625 |
| *Cenchrus pedicellatus* | 1955 | Santiago | 14.933066 | -23.523038 | 75 |
| *Cenchrus pedicellatus* | 1994 | Santiago | 15.050618 | -23.583656 | 350 |
| *Cenchrus pedicellatus* | 1994 | Santiago | 14.944175 | -23.554597 | 235 |
| *Cenchrus pedicellatus* | 1989 | Santiago | 15.083333 | -23.666667 | 388 |
| *Cenchrus pedicellatus* | 1994 | Santiago | 14.933333 | -23.566667 | 107 |
| *Cenchrus pedicellatus* | 1994 | Santiago | 15.183333 | -23.75 | 540 |
| *Cenchrus pedicellatus* | 1995 | Santiago | 15.033333 | -23.6 | 820 |
| *Cenchrus pedicellatus* | 1995 | Santiago | 15.133333 | -23.666667 | 384 |
| *Cenchrus pedicellatus* | 1995 | Santiago | 15.133333 | -23.683333 | 458 |
| *Cenchrus pedicellatus* | 1994 | Santiago | 15.116667 | -23.683333 | 498 |
| *Cenchrus pedicellatus* | 1994 | Santiago | 15.133333 | -23.683333 | 458 |
| *Cenchrus pedicellatus* | 1994 | Santiago | 15.116667 | -23.683333 | 498 |
| *Cenchrus pedicellatus* | 1994 | Santiago | 15.133333 | -23.683333 | 458 |
| *Cenchrus pedicellatus* | 1994 | Santiago | 15.15 | -23.566667 | 63 |
| *Cenchrus pedicellatus* | 1994 | Santiago | 15.15 | -23.583333 | 43 |
| *Cenchrus pedicellatus* | 1994 | Santiago | 15.15 | -23.583333 | 43 |
| *Cenchrus pedicellatus* | 1994 | Santiago | 15.016667 | -23.6 | 768 |
| *Cenchrus pedicellatus* | 1994 | Santiago | 15.033333 | -23.583333 | 506 |
| *Cenchrus pedicellatus* | 1994 | Santiago | 15.033333 | -23.616667 | 928 |
| *Cenchrus pedicellatus* | 1994 | Santiago | 15.033333 | -23.616667 | 928 |
| *Cenchrus pedicellatus* | 1994 | Santiago | 15.233333 | -23.733333 | 176 |
| *Cenchrus pedicellatus* | 1994 | Santiago | 15.2 | -23.75 | 218 |
| *Cenchrus pedicellatus* | 1994 | Santiago | 15.166667 | -23.733333 | 482 |
| *Cenchrus pedicellatus* | 1994 | Santiago | 15.233333 | -23.733333 | 176 |
| *Cenchrus pedicellatus* | 1994 | Santiago | 15.233333 | -23.733333 | 176 |
| *Cenchrus pedicellatus* | 1994 | Santiago | 15.216667 | -23.716667 | 404 |
| *Cenchrus pedicellatus* | 1994 | Santiago | 15.083333 | -23.65 | 625 |
| *Cenchrus pedicellatus* | 1994 | Santiago | 15.05 | -23.583333 | 320 |
| *Cenchrus pedicellatus* | 1994 | Santiago | 15.033333 | -23.583333 | 506 |
| *Cenchrus pedicellatus* | 1992 | Santiago | 15.216667 | -23.716667 | 404 |
| *Cenchrus polystachios* subsp. *atrichus* | 1990 | Santo Antão | 17.131584 | -25.097558 | 566 |
| *Cenchrus polystachios* subsp. *atrichus* | 1955 | Santiago | 15.175787 | -23.676035 | 985 |
| *Cenchrus polystachios* subsp. *atrichus* | 1955 | Santiago | 15.035896 | -23.661494 | 870 |
| *Digitaria ciliaris* | 1964 | Maio | 15.144031 | -23.218299 | 1 |
| *Digitaria ciliaris* | 1964 | Maio | 15.171728 | -23.179728 | 86 |
| *Digitaria ciliaris* | 1964 | Maio | 15.131595 | -23.203867 | 14 |
| *Digitaria ciliaris* | 1964 | Maio | 15.130662 | -23.184685 | 11 |
| *Digitaria ciliaris* | 1964 | Maio | 15.267055 | -23.209113 | 5 |
| *Digitaria ciliaris* | 1983 | Brava | 14.865727 | -24.697610 | 650 |
| *Digitaria ciliaris* | 1983 | Brava | 14.865742 | -24.697285 | 650 |
| *Digitaria ciliaris* | 1984 | Fogo | 15.016226 | -24.416736 | 350 |
| *Digitaria ciliaris* | 1983 | Fogo | 15.002665 | -24.441881 | 300 |
| *Digitaria ciliaris* | 1984 | Fogo | 15.022574 | -24.359301 | 940 |
| *Digitaria ciliaris* | 1984 | Fogo | 14.919992 | -24.291264 | 250 |
| *Digitaria ciliaris* | 1984 | Fogo | 15.047595 | -24.353605 | 30 |
| *Digitaria ciliaris* | 1984 | Fogo | 15.022514 | -24.359075 | 940 |
| *Digitaria ciliaris* | 1983 | Fogo | 14.979074 | -24.292646 | 100 |
| *Digitaria ciliaris* | 1983 | Santiago | 15.054124 | -23.603935 | 301 |
| *Digitaria ciliaris* | 1983 | Santiago | 15.046446 | -23.605430 | 400 |
| *Digitaria ciliaris* | 1955 | Santiago | 14.912960 | -23.482152 | 37 |
| *Digitaria ciliaris* | 1984 | Santiago | 15.080716 | -23.644767 | 421 |
| *Digitaria ciliaris* | 1955 | Santiago | 14.990349 | -23.521128 | 242 |
| *Digitaria ciliaris* | 1983 | Santiago | 15.054231 | -23.603832 | 301 |
| *Digitaria ciliaris* | 1983 | Santiago | 15.054197 | -23.603868 | 301 |
| *Digitaria ciliaris* | 1983 | Santiago | 15.117274 | -23.699448 | 550 |
| *Digitaria ciliaris* | 1982 | Santiago | 15.053160 | -23.605326 | 312 |
| *Digitaria ciliaris* | 1983 | Santiago | 15.057146 | -23.601500 | 286 |
| *Digitaria ciliaris* | 1983 | Santiago | 15.05 | -23.65 | 917 |
| *Digitaria ciliaris* | 1980 | Santiago | 15.110016 | -23.666946 | 400 |
| *Digitaria ciliaris* | 1981 | Santiago | 15.105959 | -23.669641 | 520 |
| *Digitaria ciliaris* | 1980 | Santiago | 15.103466 | -23.663036 | 540 |
| *Digitaria ciliaris* | 1981 | Santiago | 15.117827 | -23.604295 | 300 |
| *Digitaria ciliaris* | 1956 | Santo Antão | 17.148341 | -25.101673 | 310 |
| *Digitaria ciliaris* | 1956 | Santo Antão | 17.141322 | -25.029397 | 146 |
| *Digitaria ciliaris* | 1992 | Santiago | 15.215507 | -23.712181 | 465 |
| *Digitaria ciliaris* | 1994 | Santiago | 15.056077 | -23.600049 | 276 |
| *Digitaria ciliaris* | 1994 | Santiago | 15.036410 | -23.572972 | 400 |
| *Digitaria ciliaris* | 1994 | Santiago | 15.065259 | -23.624391 | 540 |
| *Digitaria ciliaris* | 1994 | Santiago | 15.233539 | -23.719222 | 265 |
| *Digitaria ciliaris* | 1994 | Santiago | 15.160761 | -23.716206 | 420 |
| *Digitaria ciliaris* | 1994 | Santiago | 15.203054 | -23.737597 | 430 |
| *Digitaria ciliaris* | 1994 | Santiago | 15.231602 | -23.743113 | 15 |
| *Digitaria ciliaris* | 1994 | Santiago | 15.033884 | -23.623936 | 999 |
| *Digitaria ciliaris* | 1994 | Santiago | 15.018193 | -23.564798 | 520 |
| *Digitaria ciliaris* | 1994 | Santiago | 15.027838 | -23.577201 | 330 |
| *Digitaria ciliaris* | 1994 | Santiago | 15.027472 | -23.577099 | 555 |
| *Digitaria ciliaris* | 1994 | Santiago | 15.027165 | -23.581091 | 590 |
| *Digitaria ciliaris* | 1994 | Santiago | 15.031406 | -23.611056 | 865 |
| *Digitaria ciliaris* | 1994 | Santiago | 15.054739 | -23.627944 | 630 |
| *Digitaria ciliaris* | 1994 | Santiago | 15.089300 | -23.672454 | 540 |
| *Digitaria ciliaris* | 1994 | Santiago | 15.065009 | -23.678098 | 680 |
| *Digitaria ciliaris* | 1994 | Santiago | 15.141285 | -23.676907 | 460 |
| *Digitaria ciliaris* | 1995 | Santiago | 15.287733 | -23.757573 | 70 |
| *Digitaria ciliaris* | 1995 | Santiago | 15.290860 | -23.759663 | 82 |
| *Digitaria ciliaris* | 1995 | Santiago | 15.141829 | -23.665089 | 220 |
| *Digitaria ciliaris* | 1995 | Santiago | 15.046351 | -23.615926 | 510 |
| *Digitaria ciliaris* | 1995 | Santiago | 15.032091 | -23.592533 | 735 |
| *Digitaria ciliaris* | 1995 | Santiago | 15.015028 | -23.587198 | 660 |
| *Digitaria ciliaris* | 1995 | Santiago | 15.070791 | -23.574931 | 165 |
| *Digitaria ciliaris* | 1995 | Santiago | 15.178636 | -23.686939 | 870 |
| *Digitaria ciliaris* | 1964 | Maio | 15.1392 | -23.1939 | 29 |
| *Digitaria ciliaris* | 1976 | Santo Antão | 17.1114 | -25.0714 | 800 |
| *Digitaria ciliaris* | 1982 | Santo Antão | 17.117 | -25.05 | 770 |
| *Digitaria eriantha* | 1994 | Santiago | 15.089525 | -23.629487 | 300 |
| *Digitaria horizontalis* | 1991 | Brava | 14.867014 | -24.699637 | 600 |
| *Digitaria horizontalis* | 1956 | Santiago | 14.948263 | -23.659253 | 48 |
| *Digitaria horizontalis* | 1989 | Santiago | 15.054040 | -23.603821 | 300 |
| *Digitaria horizontalis* | 1983 | Santiago | 14.918052 | -23.602686 | 23 |
| *Digitaria horizontalis* | 1961 | Santo Antão | 16.960963 | -25.307178 | 68 |
| *Digitaria horizontalis* | 1956 | Santo Antão | 17.113800 | -25.224565 | 250 |
| *Digitaria horizontalis* | 1894 | Santo Antão | 17.118575 | -24.998789 | 95 |
| *Digitaria horizontalis* | 1956 | São Vicente | 16.817104 | -24.963378 | 150 |
| *Digitaria horizontalis* | 1993 | Santiago | 15.181199 | -23.655296 | 400 |
| *Digitaria horizontalis* | 1993 | Santiago | 15.208489 | -23.666732 | 150 |
| *Digitaria horizontalis* | 2004 | Fogo | 14.880375 | -24.354942 | 1040 |
| *Digitaria horizontalis* | 1992 | Santiago | 15.083333 | -23.583333 | 250 |
| *Digitaria horizontalis* | 1992 | Santiago | 15.116667 | -23.55 | 28 |
| *Digitaria horizontalis* | 1992 | Santiago | 15.116667 | -23.566667 | 190 |
| *Digitaria horizontalis* | 1992 | Santiago | 15.066667 | -23.583333 | 216 |
| *Digitaria horizontalis* | 1992 | Santiago | 15.05 | -23.633333 | 1037 |
| *Digitaria horizontalis* | 1992 | Santiago | 15.066667 | -23.633333 | 552 |
| *Digitaria horizontalis* | 1992 | Santiago | 15.133333 | -23.583333 | 115 |
| *Digitaria horizontalis* | 1993 | Santiago | 15.2 | -23.666667 | 424 |
| *Digitaria horizontalis* | 1993 | Santiago | 15.166667 | -23.666667 | 528 |
| *Digitaria horizontalis* | 1993 | Santiago | 15.166667 | -23.666667 | 528 |
| *Digitaria horizontalis* | 1972 | Fogo | 14.9253 | -24.41 | 1100 |
| *Digitaria nodosa* | 1956 | Santo Antão | 17.009408 | -25.114402 | 70 |
| *Digitaria nodosa* | 1961 | Santo Antão | 16.964058 | -25.305057 | 121 |
| *Digitaria nodosa* | 1956 | Santo Antão | 16.953529 | -25.155741 | 35 |
| *Digitaria nodosa* | 1956 | Santo Antão | 17.111907 | -25.247594 | 250 |
| *Digitaria nodosa* | 1990 | Santo Antão | 17.144311 | -25.113398 | 425 |
| *Digitaria nodosa* | 1934 | Santo Antão | 17.146833 | -25.015424 | 54 |
| *Digitaria nodosa* | 1934 | Santo Antão | 17.195587 | -25.088567 | 65 |
| *Digitaria nodosa* | 1934 | Santo Antão | 17.126001 | -25.080654 | 1210 |
| *Digitaria nodosa* | 1864 | Santo Antão | 17.180630 | -25.069105 | 121 |
| *Digitaria nodosa* | 1894 | Santo Antão | 17.159792 | -25.045991 | 548 |
| *Digitaria nodosa* | 1994 | Santiago | 15.083333 | -23.65 | 625 |
| *Digitaria nuda* | 1839 | Santiago | 15.024840 | -23.557748 | 280 |
| *Digitaria nuda* | 1839 | Santiago | 15.024882 | -23.558172 | 282 |
| *Digitaria nuda* | 1992 | Santiago | 15.118611 | -23.527222 | 22 |
| *Digitaria nuda* | 1992 | Santiago | 15.121111 | -23.534444 | 60 |
| *Digitaria nuda* | 1992 | Santiago | 15.108045 | -23.528569 | 80 |
| *Digitaria nuda* | 1992 | Santiago | 15.108045 | -23.528569 | 80 |
| *Digitaria nuda* | 1992 | Santiago | 15.053650 | -23.627843 | 640 |
| *Digitaria nuda* | 1992 | Santiago | 15.060129 | -23.622981 | 480 |
| *Digitaria nuda* | 1992 | Santiago | 15.071132 | -23.567111 | 150 |
| *Digitaria nuda* | 1992 | Santiago | 15.076099 | -23.572289 | 300 |
| *Digitaria nuda* | 1992 | Santiago | 15.035443 | -23.620090 | 995 |
| *Digitaria nuda* | 1992 | Santiago | 15.036497 | -23.621792 | 1040 |
| *Digitaria nuda* | 1992 | Santiago | 15.069562 | -23.573477 | 148 |
| *Digitaria nuda* | 1992 | Santiago | 14.915249 | -23.603576 | 40 |
| *Digitaria nuda* | 1992 | Santiago | 15.010556 | -23.537222 | 340 |
| *Digitaria nuda* | 1994 | Santiago | 15.056020 | -23.600033 | 277 |
| *Digitaria nuda* | 1994 | Santiago | 15.042032 | -23.599349 | 560 |
| *Digitaria nuda* | 1994 | Santiago | 15.041936 | -23.599307 | 560 |
| *Digitaria nuda* | 1994 | Santiago | 15.049351 | -23.581878 | 280 |
| *Digitaria nuda* | 1994 | Santiago | 15.064420 | -23.612995 | 350 |
| *Digitaria nuda* | 1994 | Santiago | 15.066601 | -23.626737 | 555 |
| *Digitaria nuda* | 1994 | Santiago | 15.094020 | -23.624314 | 300 |
| *Digitaria nuda* | 1994 | Santiago | 15.094020 | -23.624228 | 300 |
| *Digitaria nuda* | 1994 | Santiago | 15.052782 | -23.606796 | 324 |
| *Digitaria nuda* | 1994 | Santiago | 15.088304 | -23.630089 | 310 |
| *Digitaria nuda* | 1994 | Santiago | 15.089526 | -23.629509 | 300 |
| *Digitaria nuda* | 1994 | Santiago | 15.088992 | -23.631936 | 330 |
| *Digitaria nuda* | 1994 | Santiago | 15.095994 | -23.627395 | 260 |
| *Digitaria nuda* | 1994 | Santiago | 15.091763 | -23.628411 | 290 |
| *Digitaria nuda* | 1994 | Santiago | 15.206797 | -23.697437 | 652 |
| *Digitaria nuda* | 1994 | Santiago | 15.216400 | -23.701972 | 510 |
| *Digitaria nuda* | 1994 | Santiago | 15.216441 | -23.701824 | 502 |
| *Digitaria nuda* | 1994 | Santiago | 15.233845 | -23.718694 | 286 |
| *Digitaria nuda* | 1994 | Santiago | 15.233717 | -23.719291 | 267 |
| *Digitaria nuda* | 1994 | Santiago | 15.283265 | -23.752627 | 20 |
| *Digitaria nuda* | 1994 | Santiago | 15.285807 | -23.753882 | 30 |
| *Digitaria nuda* | 1994 | Santiago | 15.109485 | -23.676898 | 545 |
| *Digitaria nuda* | 1994 | Santiago | 15.247668 | -23.740153 | 20 |
| *Digitaria nuda* | 1994 | Santiago | 15.243836 | -23.739893 | 32 |
| *Digitaria nuda* | 1994 | Santiago | 15.240920 | -23.740830 | 58 |
| *Digitaria nuda* | 1994 | Santiago | 15.231928 | -23.744009 | 7 |
| *Digitaria nuda* | 1994 | Santiago | 15.036502 | -23.621291 | 1033 |
| *Digitaria nuda* | 1994 | Santiago | 15.035483 | -23.621082 | 1004 |
| *Digitaria nuda* | 1994 | Santiago | 15.043318 | -23.576651 | 350 |
| *Digitaria nuda* | 1994 | Santiago | 15.018628 | -23.568864 | 400 |
| *Digitaria nuda* | 1994 | Santiago | 15.017296 | -23.569030 | 430 |
| *Digitaria nuda* | 1994 | Santiago | 15.015880 | -23.567034 | 485 |
| *Digitaria nuda* | 1994 | Santiago | 15.017284 | -23.564873 | 540 |
| *Digitaria nuda* | 1994 | Santiago | 15.020842 | -23.566429 | 370 |
| *Digitaria nuda* | 1994 | Santiago | 15.022995 | -23.566208 | 325 |
| *Digitaria nuda* | 1994 | Santiago | 15.027238 | -23.581008 | 590 |
| *Digitaria nuda* | 1994 | Santiago | 15.031800 | -23.612221 | 880 |
| *Digitaria nuda* | 1994 | Santiago | 15.029076 | -23.601619 | 800 |
| *Digitaria nuda* | 1994 | Santiago | 15.027685 | -23.607295 | 840 |
| *Digitaria nuda* | 1994 | Santiago | 15.028555 | -23.608393 | 833 |
| *Digitaria nuda* | 1994 | Santiago | 15.023347 | -23.588946 | 695 |
| *Digitaria nuda* | 1994 | Santiago | 15.162877 | -23.582875 | 60 |
| *Digitaria nuda* | 1994 | Santiago | 14.939726 | -23.553657 | 140 |
| *Digitaria nuda* | 1994 | Santiago | 14.944101 | -23.554745 | 235 |
| *Digitaria nuda* | 1994 | Santiago | 15.144978 | -23.568807 | 30 |
| *Digitaria nuda* | 1994 | Santiago | 15.064787 | -23.608055 | 310 |
| *Digitaria nuda* | 1994 | Santiago | 15.062624 | -23.620711 | 420 |
| *Digitaria nuda* | 1994 | Santiago | 15.057800 | -23.624864 | 530 |
| *Digitaria nuda* | 1994 | Santiago | 15.052929 | -23.630747 | 750 |
| *Digitaria nuda* | 1994 | Santiago | 15.052273 | -23.631160 | 777 |
| *Digitaria nuda* | 1994 | Santiago | 15.085919 | -23.674072 | 425 |
| *Digitaria nuda* | 1994 | Santiago | 15.085633 | -23.662402 | 410 |
| *Digitaria nuda* | 1994 | Santiago | 15.140878 | -23.676624 | 446 |
| *Digitaria nuda* | 1994 | Santiago | 15.141125 | -23.677363 | 456 |
| *Digitaria nuda* | 1994 | Santiago | 15.132995 | -23.667518 | 390 |
| *Digitaria nuda* | 1995 | Santiago | 15.284332 | -23.753625 | 20 |
| *Digitaria nuda* | 1995 | Santiago | 15.294654 | -23.764245 | 130 |
| *Digitaria nuda* | 1995 | Santiago | 15.293589 | -23.762893 | 43 |
| *Digitaria nuda* | 1995 | Santiago | 15.141108 | -23.664836 | 240 |
| *Digitaria nuda* | 1995 | Santiago | 15.027792 | -23.575962 | 530 |
| *Digitaria nuda* | 1995 | Santiago | 15.022540 | -23.593135 | 770 |
| *Digitaria nuda* | 1995 | Santiago | 14.995170 | -23.569880 | 390 |
| *Digitaria nuda* | 1995 | Santiago | 14.994603 | -23.570122 | 400 |
| *Digitaria nuda* | 1995 | Santiago | 14.985771 | -23.575301 | 355 |
| *Digitaria nuda* | 1995 | Santiago | 15.070715 | -23.575107 | 165 |
| *Digitaria nuda* | 1995 | Santiago | 14.982584 | -23.704432 | 65 |
| *Digitaria nuda* | 1995 | Santiago | 15.163183 | -23.577033 | 15 |
| *Digitaria nuda* | 1955 | Santiago | 14.933333 | -23.516667 | 27 |
| *Digitaria nuda* | 1992 | Santiago | 15.083333 | -23.583333 | 250 |
| *Digitaria nuda* | 1992 | Santiago | 15.116667 | -23.566667 | 190 |
| *Digitaria nuda* | 1992 | Santiago | 15.066667 | -23.583333 | 216 |
| *Digitaria nuda* | 1992 | Santiago | 15.116667 | -23.55 | 28 |
| *Digitaria nuda* | 1992 | Santiago | 15.05 | -23.633333 | 1037 |
| *Digitaria nuda* | 1992 | Santiago | 15.066667 | -23.633333 | 552 |
| *Digitaria nuda* | 1992 | Santiago | 15.133333 | -23.583333 | 115 |
| *Digitaria nuda* | 1982 | Santo Antão | 17.183 | -25.067 | 60 |
| *Digitaria nuda* | 1976 | São Nicolau | 16.6222 | -24.3356 | 750 |
| *Digitaria sanguinalis* | 1995 | Santiago | 15.178659 | -23.687029 | 870 |
| *Digitaria sanguinalis* | 2016 | Fogo | 15.014973 | -24.404802 | 598 |
| *Echinochloa colona* | 1964 | Maio | 15.128086 | -23.150474 | 12 |
| *Echinochloa colona* | 1955 | Santiago | 15.130425 | -23.528549 | 6 |
| *Echinochloa colona* | 1987 | Santiago | 15.147603 | -23.561952 | 10 |
| *Echinochloa colona* | 1984 | Santiago | 15.117820 | -23.767952 | 20 |
| *Echinochloa colona* | 1983 | Santiago | 14.937458 | -23.525145 | 45 |
| *Echinochloa colona* | 1983 | Santiago | 15.336326 | -23.740910 | 80 |
| *Echinochloa colona* | 1983 | Santiago | 15.146930 | -23.565749 | 14 |
| *Echinochloa colona* | 1981 | Santiago | 15.116100 | -23.602821 | 240 |
| *Echinochloa colona* | 1980 | Santiago | 15.110134 | -23.666920 | 400 |
| *Echinochloa colona* | 1980 | Santiago | 15.107094 | -23.670381 | 530 |
| *Echinochloa colona* | 1934 | Santiago | 15.023310 | -23.577048 | 461 |
| *Echinochloa colona* | 1934 | Santiago | 15.023447 | -23.576733 | 463 |
| *Echinochloa colona* | 1894 | Santo Antão | 17.08333 | -25.166670 | 1538 |
| *Echinochloa colona* | 1934 | Santo Antão | 17.145529 | -25.017931 | 66 |
| *Echinochloa colona* | 1956 | São Nicolau | 16.639060 | -24.316104 | 149 |
| *Echinochloa colona* | 1990 | São Vicente | 16.877425 | -24.930894 | 510 |
| *Echinochloa colona* | 1992 | Santiago | 15.121111 | -23.533889 | 60 |
| *Echinochloa colona* | 1992 | Santiago | 15.121389 | -23.535000 | 59 |
| *Echinochloa colona* | 1992 | Santiago | 15.110186 | -23.525798 | 20 |
| *Echinochloa colona* | 1994 | Santiago | 15.089458 | -23.629545 | 300 |
| *Echinochloa colona* | 1994 | Santiago | 15.091861 | -23.628440 | 290 |
| *Echinochloa colona* | 1994 | Santiago | 15.230682 | -23.742300 | 20 |
| *Echinochloa colona* | 1994 | Santiago | 15.164400 | -23.582391 | 32 |
| *Echinochloa colona* | 1994 | Santiago | 15.143568 | -23.569021 | 20 |
| *Echinochloa colona* | 1995 | Santiago | 15.262393 | -23.746395 | 10 |
| *Echinochloa colona* | 1995 | Santiago | 15.163249 | -23.576611 | 15 |
| *Echinochloa colona* | 1992 | Santiago | 15.116667 | -23.566667 | 190 |
| *Echinochloa colona* | 1992 | Santiago | 15.116667 | -23.55 | 28 |
| *Echinochloa colona* | 1992 | Santiago | 15.1 | -23.533333 | 30 |
| *Echinochloa colona* | 1992 | Santiago | 15.133333 | -23.55 | 82 |
| *Echinochloa colona* | 1992 | Santiago | 15.133333 | -23.566667 | 46 |
| *Echinochloa colona* | 1992 | Santiago | 15.133333 | -23.583333 | 115 |
| *Echinochloa colona* | 1995 | Santiago | 15.15 | -23.566667 | 63 |
| *Echinochloa colona* | 1995 | Santiago | 14.983333 | -23.716667 | 43 |
| *Echinochloa colona* | 1995 | Santiago | 15.266667 | -23.75 | 11 |
| *Echinochloa colona* | 1994 | Santiago | 15.166667 | -23.6 | 75 |
| *Echinochloa colona* | 1994 | Santiago | 15.083333 | -23.65 | 625 |
| *Echinochloa colona* | 1994 | Santiago | 15.083333 | -23.65 | 625 |
| *Echinochloa colona* | 1992 | Santiago | 15.1 | -23.55 | 94 |
| *Echinochloa colona* | 1992 | Santiago | 15.1 | -23.55 | 94 |
| *Echinochloa colona* | 1976 | Santo Antão | 17.1461 | -25.0689 | 213 |
| *Echinochloa colona* | 1976 | São Vicente | 16.8717 | -24.9331 | 750 |
| *Eleusine indica* | 1983 | Brava | 14.865721 | -24.697442 | 650 |
| *Eleusine indica* | 1983 | Brava | 14.865482 | -24.697439 | 652 |
| *Eleusine indica* | 1956 | Brava | 14.855505 | -24.686618 | 590 |
| *Eleusine indica* | 1991 | Fogo | 14.982465 | -24.458368 | 430 |
| *Eleusine indica* | 1983 | Fogo | 15.002574 | -24.441075 | 300 |
| *Eleusine indica* | 1984 | Fogo | 15.015271 | -24.417388 | 350 |
| *Eleusine indica* | 1987 | Santiago | 15.052420 | -23.605121 | 325 |
| *Eleusine indica* | 1955 | Santiago | 14.954343 | -23.559291 | 161 |
| *Eleusine indica* | 1955 | Santiago | 14.954687 | -23.560631 | 165 |
| *Eleusine indica* | 1982 | Santiago | 15.052000 | -23.608237 | 334 |
| *Eleusine indica* | 1982 | Santiago | 15.052271 | -23.603245 | 315 |
| *Eleusine indica* | 1982 | Santiago | 15.145315 | -23.565136 | 24 |
| *Eleusine indica* | 1987 | Santiago | 15.052815 | -23.604465 | 316 |
| *Eleusine indica* | 1989 | Santiago | 15.053614 | -23.602527 | 296 |
| *Eleusine indica* | 1983 | Santiago | 15.146834 | -23.565792 | 14 |
| *Eleusine indica* | 1983 | Santiago | 15.057076 | -23.601436 | 285 |
| *Eleusine indica* | 1934 | Santiago | 14.934965 | -23.518358 | 30 |
| *Eleusine indica* | 1934 | Santiago | 15.022985 | -23.576947 | 456 |
| *Eleusine indica* | 1934 | Santiago | 14.935032 | -23.518169 | 30 |
| *Eleusine indica* | 1908 | Santiago | 14.961388 | -23.564148 | 203 |
| *Eleusine indica* | 1893 | Santo Antão | 17.199670 | -25.090630 | 33 |
| *Eleusine indica* | 1956 | Santo Antão | 17.149616 | -25.104898 | 218 |
| *Eleusine indica* | 1961 | Santo Antão | 16.960869 | -25.307195 | 68 |
| *Eleusine indica* | 1893 | Santo Antão | 17.199670 | -25.090630 | 33 |
| *Eleusine indica* | 1956 | Santo Antão | 17.105968 | -25.242363 | 313 |
| *Eleusine indica* | 1956 | Santo Antão | 16.961895 | -25.307024 | 82 |
| *Eleusine indica* | 1990 | Santo Antão | 17.110926 | -25.157145 | 670 |
| *Eleusine indica* | 1893 | Santo Antão | 17.194740 | -25.086104 | 65 |
| *Eleusine indica* | 1992 | Santiago | 15.121111 | -23.533889 | 60 |
| *Eleusine indica* | 1992 | Santiago | 15.110186 | -23.525798 | 20 |
| *Eleusine indica* | 1992 | Santiago | 15.111352 | -23.525053 | 20 |
| *Eleusine indica* | 1992 | Santiago | 15.111352 | -23.525053 | 20 |
| *Eleusine indica* | 1994 | Santiago | 15.036387 | -23.621382 | 1033 |
| *Eleusine indica* | 1994 | Santiago | 15.022958 | -23.566311 | 325 |
| *Eleusine indica* | 1994 | Santiago | 15.037201 | -23.687132 | 485 |
| *Eleusine indica* | 2004 | Santiago | 14.940557 | -23.488077 | 90 |
| *Eleusine indica* | 2004 | Santiago | 14.913382 | -23.497898 | 25 |
| *Eleusine indica* | 2004 | São Vicente | 16.889418 | -24.988885 | 10 |
| *Eleusine indica* | 2004 | São Vicente | 16.871415 | -24.933990 | 710 |
| *Eleusine indica* | 2004 | São Nicolau | 16.617207 | -24.298783 | 164 |
| *Eleusine indica* | 2005 | Santo Antão | 17.177617 | -25.064487 | 22 |
| *Eleusine indica* | 2005 | Santiago | 15.101101 | -23.672372 | 580 |
| *Eleusine indica* | 2006 | Santiago | 15.183926 | -23.688473 | 750 |
| *Eleusine indica* | 2006 | Fogo | 14.921132 | -24.487626 | 255 |
| *Eleusine indica* | 1989 | Santiago | 15.083333 | -23.666667 | 388 |
| *Eleusine indica* | 1982 | Brava | 14.825 | -24.725 | 291 |
| *Eleusine indica* | 1982 | Santo Antão | 16.96 | -25.31 | 31 |
| *Eleusine indica* | 1992 | Santiago | 15.116667 | -23.566667 | 190 |
| *Eleusine indica* | 1992 | Santiago | 15.1 | -23.533333 | 30 |
| *Eleusine indica* | 1992 | Santiago | 15.133333 | -23.55 | 82 |
| *Eleusine indica* | 1992 | Santiago | 15.133333 | -23.583333 | 115 |
| *Eleusine indica* | 1992 | Santiago | 15.133333 | -23.566667 | 46 |
| *Eleusine indica* | 1992 | Santiago | 15.066667 | -23.633333 | 552 |
| *Eleusine indica* | 1995 | Santiago | 15.033333 | -23.616667 | 928 |
| *Eleusine indica* | 1994 | Santiago | 15.033333 | -23.683333 | 509 |
| *Eleusine indica* | 1994 | Santiago | 15.166667 | -23.583333 | 85 |
| *Eleusine indica* | 1994 | Santiago | 15.033333 | -23.6 | 820 |
| *Eleusine indica* | 1994 | Santiago | 15.016667 | -23.583333 | 623 |
| *Eleusine indica* | 1994 | Santiago | 15.033333 | -23.633333 | 805 |
| *Eleusine indica* | 1994 | Santiago | 15.033333 | -23.633333 | 805 |
| *Eleusine indica* | 1994 | Santiago | 15.216667 | -23.716667 | 404 |
| *Eleusine indica* | 1994 | Santiago | 15.233333 | -23.733333 | 176 |
| *Eleusine indica* | 1994 | Santiago | 15.083333 | -23.65 | 625 |
| *Eleusine indica* | 1994 | Santiago | 15.083333 | -23.65 | 625 |
| *Eleusine indica* | 1994 | Santiago | 15.083333 | -23.65 | 625 |
| *Eleusine indica* | 1994 | Santiago | 15.083333 | -23.65 | 625 |
| *Eleusine indica* | 1994 | Santiago | 15.033333 | -23.616667 | 928 |
| *Eleusine indica* | 1993 | Santiago | 15.033333 | -23.633333 | 805 |
| *Eleusine indica* | 1993 | Santiago | 15.2 | -23.666667 | 424 |
| *Eleusine indica* | 1992 | Santiago | 15.066667 | -23.633333 | 552 |
| *Eleusine indica* | 1992 | Santiago | 15.216667 | -23.716667 | 404 |
| *Eleusine indica* | 1992 | Santiago | 15.1 | -23.55 | 94 |
| *Eleusine indica* | 1992 | Santiago | 15.1 | -23.55 | 94 |
| *Eleusine indica* | 1992 | Santiago | 15.05 | -23.633333 | 1037 |
| *Eleusine indica* | 1976 | São Vicente | 16.8717 | -24.9331 | 750 |
| *Eleusine indica* | 1982 | Santo Antão | 16.966667 | -25.3 | 232 |
| *Eragrostis cilianensis* | 1987 | Maio | 15.255350 | -23.203187 | 12 |
| *Eragrostis cilianensis* | 1988 | Maio | 15.252441 | -23.150332 | 40 |
| *Eragrostis cilianensis* | 1964 | Maio | 15.137859 | -23.207220 | 28 |
| *Eragrostis cilianensis* | 1964 | Maio | 15.142032 | -23.214242 | 1 |
| *Eragrostis cilianensis* | 1964 | Maio | 15.280908 | -23.136873 | 120 |
| *Eragrostis cilianensis* | 1988 | Boavista | 16.115334 | -22.882022 | 43 |
| *Eragrostis cilianensis* | 1983 | Brava | 14.865740 | -24.697340 | 651 |
| *Eragrostis cilianensis* | 1934 | Fogo | 14.886848 | -24.347756 | 1135 |
| *Eragrostis cilianensis* | 1961 | Santiago | 15.007344 | -23.522875 | 252 |
| *Eragrostis cilianensis* | 1961 | Santiago | 15.007156 | -23.523528 | 258 |
| *Eragrostis cilianensis* | 1961 | Santiago | 15.007385 | -23.523018 | 253 |
| *Eragrostis cilianensis* | 1955 | Santiago | 14.996075 | -23.518994 | 250 |
| *Eragrostis cilianensis* | 1983 | Santiago | 15.139527 | -23.750297 | 53 |
| *Eragrostis cilianensis* | 1983 | Santiago | 15.141060 | -23.596697 | 115 |
| *Eragrostis cilianensis* | 1984 | Santiago | 15.050030 | -23.603645 | 346 |
| *Eragrostis cilianensis* | 1955 | Santiago | 14.933347 | -23.531073 | 79 |
| *Eragrostis cilianensis* | 1934 | Santiago | 14.959415 | -23.561909 | 206 |
| *Eragrostis cilianensis* | 1934 | Santiago | 14.960395 | -23.561544 | 224 |
| *Eragrostis cilianensis* | 1893 | Santo Antão | 17.189948 | -25.110535 | 127 |
| *Eragrostis cilianensis* | 1893 | Santo Antão | 17.083330 | -25.166670 | 1538 |
| *Eragrostis cilianensis* | 1893 | Santo Antão | 17.190231 | -25.110745 | 120 |
| *Eragrostis cilianensis* | 1934 | Santo Antão | 17.108360 | -25.067454 | 1335 |
| *Eragrostis cilianensis* | 1934 | Santo Antão | 17.147185 | -25.018506 | 41 |
| *Eragrostis cilianensis* | 1934 | Santo Antão | 17.147050 | -25.015364 | 46 |
| *Eragrostis cilianensis* | 1934 | Santo Antão | 17.147192 | -25.018488 | 40 |
| *Eragrostis cilianensis* | 1934 | Santo Antão | 17.148909 | -25.016908 | 32 |
| *Eragrostis cilianensis* | 1934 | Santo Antão | 17.126039 | -25.080848 | 1213 |
| *Eragrostis cilianensis* | 1929 | São Nicolau | 16.606891 | -24.414579 | 25 |
| *Eragrostis cilianensis* | 1990 | São Vicente | 16.859842 | -25.047713 | 150 |
| *Eragrostis cilianensis* | 1934 | São Vicente | 16.869416 | -24.934108 | 615 |
| *Eragrostis cilianensis* | 1994 | Santiago | 15.233534 | -23.719365 | 260 |
| *Eragrostis cilianensis* | 1994 | Santiago | 15.240720 | -23.740851 | 58 |
| *Eragrostis cilianensis* | 1995 | Santiago | 15.290699 | -23.759696 | 80 |
| *Eragrostis cilianensis* | 1995 | Santiago | 15.042339 | -23.708126 | 560 |
| *Eragrostis cilianensis* | 1995 | Santiago | 14.995294 | -23.569492 | 390 |
| *Eragrostis cilianensis* | 1995 | Santiago | 14.994736 | -23.570219 | 400 |
| *Eragrostis cilianensis* | 1995 | Santiago | 14.985610 | -23.575446 | 355 |
| *Eragrostis cilianensis* | 1995 | Santiago | 14.966393 | -23.701328 | 55 |
| *Eragrostis cilianensis* | 1996 | Santiago | 14.976604 | -23.710800 | 20 |
| *Eragrostis cilianensis* | 1995 | Santiago | 14.988253 | -23.704831 | 120 |
| *Eragrostis cilianensis* | 1996 | Santiago | 14.966667 | -23.5 | 115 |
| *Eragrostis cilianensis* | 1995 | Santiago | 15.05 | -23.583333 | 320 |
| *Eragrostis cilianensis* | 1995 | Santiago | 14.95 | -23.566667 | 194 |
| *Eragrostis cilianensis* | 1995 | Santiago | 15.0 | -23.583333 | 500 |
| *Eragrostis cilianensis* | 1995 | Santiago | 14.983333 | -23.583333 | 386 |
| *Eragrostis cilianensis* | 1995 | Santiago | 15.0 | -23.583333 | 500 |
| *Eragrostis cilianensis* | 1995 | Santiago | 15.0 | -23.583333 | 500 |
| *Eragrostis cilianensis* | 1995 | Santiago | 15.0 | -23.583333 | 500 |
| *Eragrostis cilianensis* | 1995 | Santiago | 15.033333 | -23.716667 | 362 |
| *Eragrostis cilianensis* | 1994 | Santiago | 15.033333 | -23.7 | 480 |
| *Eragrostis cilianensis* | 1994 | Santiago | 15.033333 | -23.683333 | 509 |
| *Eragrostis cilianensis* | 1994 | Santiago | 15.05 | -23.633333 | 1037 |
| *Eragrostis cilianensis* | 1994 | Santiago | 15.233333 | -23.733333 | 176 |
| *Eragrostis cilianensis* | 1976 | São Vicente | 16.8806 | -24.9289 | 200 |
| *Eragrostis cilianensis* | 1982 | São Nicolau | 16.6 | -24.3 | 120 |
| *Eragrostis cilianensis* | 1964 | Maio | 15.1383 | -23.2106 | 27 |
| *Eragrostis ciliaris* | 1964 | Maio | 15.269083 | -23.112319 | 15 |
| *Eragrostis ciliaris* | 1964 | Maio | 15.130959 | -23.202570 | 13 |
| *Eragrostis ciliaris* | 1964 | Maio | 15.280630 | -23.136885 | 120 |
| *Eragrostis ciliaris* | 1964 | Maio | 15.143183 | -23.213905 | 2 |
| *Eragrostis ciliaris* | 1964 | Maio | 15.129870 | -23.183016 | 24 |
| *Eragrostis ciliaris* | 1987 | Maio | 15.177344 | -23.221381 | 11 |
| *Eragrostis ciliaris* | 1934 | Fogo | 14.871208 | -24.356936 | 880 |
| *Eragrostis ciliaris* | 1934 | Fogo | 14.922619 | -24.358844 | 1780 |
| *Eragrostis ciliaris* | 1934 | Fogo | 14.903268 | -24.461514 | 480 |
| *Eragrostis ciliaris* | 1984 | Fogo | 14.898735 | -24.292871 | 500 |
| *Eragrostis ciliaris* | 1956 | Fogo | 14.983240 | -24.434195 | 980 |
| *Eragrostis ciliaris* | 1983 | Fogo | 15.002645 | -24.442196 | 300 |
| *Eragrostis ciliaris* | 1956 | Fogo | 14.993545 | -24.409118 | 1060 |
| *Eragrostis ciliaris* | 1983 | Fogo | 14.940928 | -24.298096 | 300 |
| *Eragrostis ciliaris* | 1956 | Fogo | 14.865757 | -24.314036 | 495 |
| *Eragrostis ciliaris* | 1984 | Fogo | 15.022295 | -24.357399 | 950 |
| *Eragrostis ciliaris* | 1984 | Fogo | 15.047675 | -24.354300 | 30 |
| *Eragrostis ciliaris* | 1938 | Fogo | 14.941854 | -24.461436 | 595 |
| *Eragrostis ciliaris* | 1839 | Santiago | 15.030111 | -23.567968 | 321 |
| *Eragrostis ciliaris* | 1955 | Santiago | 15.281215 | -23.734189 | 90 |
| *Eragrostis ciliaris* | 1983 | Santiago | 15.047741 | -23.598882 | 385 |
| *Eragrostis ciliaris* | 1987 | Santiago | 15.053009 | -23.598843 | 350 |
| *Eragrostis ciliaris* | 1955 | Santiago | 14.942564 | -23.600943 | 180 |
| *Eragrostis ciliaris* | 1893 | Santo Antão | 17.160576 | -25.046127 | 543 |
| *Eragrostis ciliaris* | 1934 | Santo Antão | 17.145338 | -25.017469 | 86 |
| *Eragrostis ciliaris* | 1934 | Santo Antão | 17.145347 | -25.017434 | 86 |
| *Eragrostis ciliaris* | 1993 | Santo Antão | 17.184232 | -25.104808 | 400 |
| *Eragrostis ciliaris* | 1993 | Santo Antão | 17.014512 | -25.101140 | 45 |
| *Eragrostis ciliaris* | 1894 | Santo Antão | 17.083330 | -25.166670 | 1538 |
| *Eragrostis ciliaris* | 1894 | Santo Antão | 17.083330 | -25.166670 | 1538 |
| *Eragrostis ciliaris* | 1893 | Santo Antão | 17.160295 | -25.045978 | 543 |
| *Eragrostis ciliaris* | 1994 | Santiago | 15.055913 | -23.600126 | 277 |
| *Eragrostis ciliaris* | 1994 | Santiago | 15.049269 | -23.581877 | 280 |
| *Eragrostis ciliaris* | 1995 | Santiago | 15.294569 | -23.764447 | 130 |
| *Eragrostis ciliaris* | 1994 | Santiago | 15.033333 | -23.7 | 480 |
| *Eragrostis ciliaris* | 1994 | Santiago | 15.05 | -23.583333 | 320 |
| *Eragrostis ciliaris* | 1994 | Santiago | 15.05 | -23.616667 | 434 |
| *Eragrostis ciliaris* | 1981 | Santiago | 15.033 | -23.6 | 300 |
| *Eragrostis ciliaris* | 1982 | Fogo | 15.033 | -24.35 | 40 |
| *Eragrostis ciliaris* | 1964 | Maio | 15.25 | -23.11 | 32 |
| *Eragrostis ciliaris* | 1982 | Fogo | 15.0 | -24.4 | 940 |
| *Eragrostis ciliaris* | 1982 | Fogo | 15.0 | -24.4 | 940 |
| *Eragrostis ciliaris* | 1982 | Fogo | 15.0 | -24.4 | 940 |
| *Eragrostis ciliaris* | 1982 | São Nicolau | 16.6 | -24.3 | 125 |
| *Hordeum vulgare* | 1956 | Santo Antão | 17.086549 | -25.134127 | 1212 |
| *Hordeum vulgare* | 1956 | Santo Antão | 17.088566 | -25.150635 | 1400 |
| *Imperata cylindrica* | 1984 | Fogo | 15.001082 | -24.347206 | 1560 |
| *Imperata cylindrica* | 1934 | Fogo | 14.964116 | -24.380782 | 1740 |
| *Imperata cylindrica* | 1956 | Fogo | 15.019626 | -24.346844 | 910 |
| *Panicum laetum* | 1989 | Santiago | 14.916667 | -23.516667 | 18 |
| *Paspalum scrobiculatum* | 1956 | Brava | 14.886074 | -24.706245 | 342 |
| *Paspalum scrobiculatum* | 1984 | Fogo | 15.015454 | -24.417337 | 350 |
| *Paspalum scrobiculatum* | 1983 | Fogo | 15.001026 | -24.445067 | 300 |
| *Paspalum scrobiculatum* | 1934 | Fogo | 14.891773 | -24.294657 | 500 |
| *Paspalum scrobiculatum* | 1983 | Santiago | 15.048652 | -23.599430 | 370 |
| *Paspalum scrobiculatum* | 1955 | Santiago | 14.942477 | -23.601072 | 180 |
| *Paspalum scrobiculatum* | 1983 | Santiago | 15.053413 | -23.605080 | 308 |
| *Paspalum scrobiculatum* | 1934 | Santiago | 15.043117 | -23.635725 | 956 |
| *Paspalum scrobiculatum* | 1839 | Santiago | 15.029891 | -23.567783 | 320 |
| *Paspalum scrobiculatum* | 1994 | Santiago | 15.029231 | -23.601416 | 800 |
| *Paspalum scrobiculatum* | 1994 | Santiago | 15.143685 | -23.568832 | 20 |
| *Paspalum scrobiculatum* | 1994 | Santiago | 15.033333 | -23.6 | 820 |
| *Paspalum scrobiculatum* | 1972 | Santiago | 15.11 | -23.6397 | 730 |
| *Paspalum scrobiculatum* | 1976 | Santiago | 15.1817 | -23.6781 | 900 |
| *Setaria pumila* | 1987 | Maio | 15.248698 | -23.206581 | 10 |
| *Setaria pumila* | 1964 | Maio | 15.266811 | -23.209013 | 5 |
| *Setaria pumila* | 1983 | Brava | 14.865742 | -24.697312 | 649 |
| *Setaria pumila* | 1983 | Fogo | 15.042563 | -24.362970 | 400 |
| *Setaria pumila* | 1984 | Fogo | 15.014299 | -24.336755 | 900 |
| *Setaria pumila* | 1934 | Fogo | 14.941487 | -24.459244 | 640 |
| *Setaria pumila* | 1955 | Santiago | 15.175690 | -23.676003 | 985 |
| *Setaria pumila* | 1984 | Santiago | 15.047842 | -23.603776 | 368 |
| *Setaria pumila* | 1981 | Santiago | 15.113388 | -23.656793 | 530 |
| *Setaria pumila* | 1934 | Santo Antão | 17.107577 | -25.067652 | 1350 |
| *Setaria pumila* | 1934 | Santo Antão | 17.110959 | -25.062516 | 1205 |
| *Setaria pumila* | 1934 | Santo Antão | 17.145327 | -25.017320 | 88 |
| *Setaria pumila* | 1934 | Santo Antão | 17.105317 | -25.062756 | 1180 |
| *Setaria pumila* | 1934 | Santo Antão | 17.108571 | -25.059478 | 1185 |
| *Setaria pumila* | 1992 | Santiago | 15.035318 | -23.620147 | 990 |
| *Setaria pumila* | 1994 | Santiago | 15.088157 | -23.630991 | 310 |
| *Setaria pumila* | 1994 | Santiago | 15.206750 | -23.697341 | 650 |
| *Setaria pumila* | 1994 | Santiago | 15.203172 | -23.737534 | 430 |
| *Setaria pumila* | 1994 | Santiago | 15.027384 | -23.578760 | 575 |
| *Setaria pumila* | 1994 | Santiago | 15.023190 | -23.589024 | 700 |
| *Setaria pumila* | 1994 | Santiago | 15.077884 | -23.670391 | 445 |
| *Setaria pumila* | 1995 | Santiago | 15.044446 | -23.616115 | 530 |
| *Setaria pumila* | 1995 | Santiago | 15.027766 | -23.575921 | 530 |
| *Setaria pumila* | 1995 | Santiago | 15.177966 | -23.684230 | 845 |
| *Setaria pumila* | 2004 | Fogo | 15.015929 | -24.427488 | 165 |
| *Setaria pumila* | 2006 | Santiago | 15.036119 | -23.620880 | 1025 |
| *Setaria pumila* | 2016 | Fogo | 15.014954 | -24.404824 | 539 |
| *Setaria pumila* | 1995 | Santiago | 15.183333 | -23.7 | 780 |
| *Setaria pumila* | 1995 | Santiago | 15.0 | -23.7 | 389 |
| *Setaria pumila* | 1995 | Santiago | 15.0 | -23.583333 | 500 |
| *Setaria pumila* | 1995 | Santiago | 15.033333 | -23.6 | 820 |
| *Setaria pumila* | 1995 | Santiago | 15.033333 | -23.583333 | 506 |
| *Setaria pumila* | 1995 | Santiago | 15.016667 | -23.583333 | 623 |
| *Setaria pumila* | 1997 | Santiago | 15.033333 | -23.616667 | 928 |
| *Setaria pumila* | 1995 | Santiago | 15.066667 | -23.7 | 608 |
| *Setaria pumila* | 1994 | Santiago | 15.133333 | -23.683333 | 458 |
| *Setaria pumila* | 1994 | Santiago | 15.15 | -23.583333 | 43 |
| *Setaria pumila* | 1994 | Santiago | 15.016667 | -23.6 | 768 |
| *Setaria pumila* | 1994 | Santiago | 15.016667 | -23.6 | 768 |
| *Setaria pumila* | 1994 | Santiago | 15.033333 | -23.616667 | 928 |
| *Setaria pumila* | 1994 | Santiago | 15.033333 | -23.6 | 820 |
| *Setaria pumila* | 1994 | Santiago | 15.033333 | -23.616667 | 928 |
| *Setaria pumila* | 1994 | Santiago | 15.016667 | -23.6 | 768 |
| *Setaria pumila* | 1994 | Santiago | 15.033333 | -23.583333 | 506 |
| *Setaria pumila* | 1994 | Santiago | 15.016667 | -23.583333 | 623 |
| *Setaria pumila* | 1994 | Santiago | 15.033333 | -23.583333 | 506 |
| *Setaria pumila* | 1994 | Santiago | 15.033333 | -23.583333 | 506 |
| *Setaria pumila* | 1994 | Santiago | 15.033333 | -23.616667 | 928 |
| *Setaria pumila* | 1994 | Santiago | 15.033333 | -23.633333 | 805 |
| *Setaria pumila* | 1994 | Santiago | 15.033333 | -23.633333 | 805 |
| *Setaria pumila* | 1994 | Santiago | 15.033333 | -23.633333 | 805 |
| *Setaria pumila* | 1994 | Santiago | 15.2 | -23.75 | 218 |
| *Setaria pumila* | 1994 | Santiago | 15.216667 | -23.716667 | 404 |
| *Setaria pumila* | 1994 | Santiago | 15.166667 | -23.7 | 533 |
| *Setaria pumila* | 1994 | Santiago | 15.216667 | -23.716667 | 404 |
| *Setaria pumila* | 1994 | Santiago | 15.083333 | -23.65 | 625 |
| *Setaria pumila* | 1992 | Santiago | 15.033333 | -23.633333 | 805 |
| *Setaria pumila* | 1992 | Santiago | 15.016667 | -23.633333 | 699 |
| *Setaria pumila* | 1964 | Maio | 15.1525 | -23.2169 | 1 |
| *Setaria verticillata* | 1964 | Maio | 15.135906 | -23.207819 | 25 |
| *Setaria verticillata* | 1964 | Maio | 15.280422 | -23.137017 | 112 |
| *Setaria verticillata* | 1964 | Maio | 15.129240 | -23.150074 | 8 |
| *Setaria verticillata* | 1964 | Maio | 15.184801 | -23.217857 | 14 |
| *Setaria verticillata* | 1956 | Boavista | 16.139528 | -22.725715 | 17 |
| *Setaria verticillata* | 1983 | Brava | 14.843093 | -24.714014 | 680 |
| *Setaria verticillata* | 1956 | Brava | 14.836553 | -24.694132 | 596 |
| *Setaria verticillata* | 1984 | Brava | 14.885396 | -24.717344 | 30 |
| *Setaria verticillata* | 1982 | Brava | 14.871161 | -24.695641 | 496 |
| *Setaria verticillata* | 1983 | Fogo | 15.001587 | -24.444459 | 300 |
| *Setaria verticillata* | 1984 | Fogo | 15.021996 | -24.357586 | 960 |
| *Setaria verticillata* | 1956 | Fogo | 14.908143 | -24.451281 | 600 |
| *Setaria verticillata* | 1938 | Fogo | 14.939001 | -24.457876 | 620 |
| *Setaria verticillata* | 1908 | Fogo | 14.875594 | -24.425792 | 575 |
| *Setaria verticillata* | 1987 | Santiago | 14.957785 | -23.516307 | 125 |
| *Setaria verticillata* | 1983 | Santiago | 15.053937 | -23.604238 | 302 |
| *Setaria verticillata* | 1982 | Santiago | 15.051765 | -23.608127 | 340 |
| *Setaria verticillata* | 1955 | Santiago | 14.933372 | -23.523169 | 66 |
| *Setaria verticillata* | 1982 | Santiago | 15.145449 | -23.565279 | 20 |
| *Setaria verticillata* | 1983 | Santiago | 15.094088 | -23.622974 | 295 |
| *Setaria verticillata* | 1983 | Santiago | 15.057134 | -23.601395 | 283 |
| *Setaria verticillata* | 1980 | Santiago | 15.107177 | -23.670145 | 524 |
| *Setaria verticillata* | 1961 | Santo Antão | 16.972388 | -25.290269 | 346 |
| *Setaria verticillata* | 1893 | Santo Antão | 17.083330 | -25.166670 | 1538 |
| *Setaria verticillata* | 1956 | Santo Antão | 17.141466 | -25.029590 | 147 |
| *Setaria verticillata* | 1990 | Santo Antão | 17.136915 | -25.065639 | 310 |
| *Setaria verticillata* | 1934 | Santo Antão | 17.148897 | -25.016813 | 28 |
| *Setaria verticillata* | 1893 | Santo Antão | 17.083330 | -25.166670 | 1538 |
| *Setaria verticillata* | 1934 | São Vicente | 16.870378 | -24.933320 | 680 |
| *Setaria verticillata* | 1992 | Santiago | 15.069167 | -23.570556 | 155 |
| *Setaria verticillata* | 1992 | Santiago | 15.118611 | -23.527222 | 22 |
| *Setaria verticillata* | 1992 | Santiago | 15.060129 | -23.622981 | 480 |
| *Setaria verticillata* | 1992 | Santiago | 15.071132 | -23.567111 | 150 |
| *Setaria verticillata* | 1994 | Santiago | 15.240785 | -23.740836 | 58 |
| *Setaria verticillata* | 1995 | Santiago | 15.259438 | -23.748640 | 5 |
| *Setaria verticillata* | 2004 | Santiago | 14.971340 | -23.462656 | 5 |
| *Setaria verticillata* | 2004 | Santiago | 14.973769 | -23.461349 | 10 |
| *Setaria verticillata* | 2004 | Fogo | 14.947844 | -24.478883 | 330 |
| *Setaria verticillata* | 2004 | Fogo | 15.016250 | -24.427571 | 160 |
| *Setaria verticillata* | 2004 | São Vicente | 16.870781 | -24.941388 | 297 |
| *Setaria verticillata* | 2004 | São Vicente | 16.871222 | -24.940383 | 310 |
| *Setaria verticillata* | 2005 | Santiago | 15.055032 | -23.481264 | 25 |
| *Setaria verticillata* | 2005 | Santiago | 15.054549 | -23.481963 | 23 |
| *Setaria verticillata* | 2007 | São Nicolau | 16.623599 | -24.350443 | 1048 |
| *Setaria verticillata* | 2007 | São Nicolau | 16.623556 | -24.350395 | 1048 |
| *Setaria verticillata* | 2007 | São Nicolau | 16.62359 | -24.350403 | 1048 |
| *Setaria verticillata* | 1989 | Santiago | 14.916667 | -23.516667 | 18 |
| *Setaria verticillata* | 1992 | Santiago | 15.083333 | -23.583333 | 250 |
| *Setaria verticillata* | 1992 | Santiago | 15.066667 | -23.566667 | 168 |
| *Setaria verticillata* | 1992 | Santiago | 15.133333 | -23.566667 | 46 |
| *Setaria verticillata* | 1992 | Santiago | 15.1 | -23.533333 | 30 |
| *Setaria verticillata* | 1992 | Santiago | 15.066667 | -23.583333 | 216 |
| *Setaria verticillata* | 1992 | Santiago | 15.133333 | -23.55 | 82 |
| *Setaria verticillata* | 1992 | Santiago | 15.066667 | -23.633333 | 552 |
| *Setaria verticillata* | 1992 | Santiago | 15.133333 | -23.583333 | 115 |
| *Setaria verticillata* | 1994 | Santiago | 14.933333 | -23.566667 | 107 |
| *Setaria verticillata* | 1995 | Santiago | 15.0 | -23.583333 | 500 |
| *Setaria verticillata* | 1995 | Santiago | 15.033333 | -23.583333 | 506 |
| *Setaria verticillata* | 1995 | Santiago | 15.0 | -23.583333 | 500 |
| *Setaria verticillata* | 1995 | Santiago | 14.983333 | -23.583333 | 386 |
| *Setaria verticillata* | 1995 | Santiago | 15.016667 | -23.583333 | 623 |
| *Setaria verticillata* | 1995 | Santiago | 15.0 | -23.583333 | 500 |
| *Setaria verticillata* | 1995 | Santiago | 15.0 | -23.583333 | 500 |
| *Setaria verticillata* | 1995 | Santiago | 15.133333 | -23.666667 | 384 |
| *Setaria verticillata* | 1995 | Santiago | 15.266667 | -23.75 | 11 |
| *Setaria verticillata* | 1994 | Santiago | 15.133333 | -23.683333 | 458 |
| *Setaria verticillata* | 1994 | Santiago | 15.133333 | -23.683333 | 458 |
| *Setaria verticillata* | 1994 | Santiago | 15.15 | -23.583333 | 43 |
| *Setaria verticillata* | 1994 | Santiago | 15.15 | -23.566667 | 63 |
| *Setaria verticillata* | 1994 | Santiago | 15.15 | -23.566667 | 63 |
| *Setaria verticillata* | 1994 | Santiago | 15.166667 | -23.6 | 75 |
| *Setaria verticillata* | 1994 | Santiago | 15.166667 | -23.583333 | 85 |
| *Setaria verticillata* | 1994 | Santiago | 15.166667 | -23.583333 | 85 |
| *Setaria verticillata* | 1994 | Santiago | 15.016667 | -23.6 | 768 |
| *Setaria verticillata* | 1994 | Santiago | 15.016667 | -23.6 | 768 |
| *Setaria verticillata* | 1994 | Santiago | 15.016667 | -23.6 | 768 |
| *Setaria verticillata* | 1994 | Santiago | 15.033333 | -23.6 | 820 |
| *Setaria verticillata* | 1994 | Santiago | 15.016667 | -23.6 | 768 |
| *Setaria verticillata* | 1994 | Santiago | 15.016667 | -23.583333 | 623 |
| *Setaria verticillata* | 1994 | Santiago | 15.016667 | -23.583333 | 623 |
| *Setaria verticillata* | 1994 | Santiago | 15.016667 | -23.6 | 768 |
| *Setaria verticillata* | 1994 | Santiago | 15.033333 | -23.633333 | 805 |
| *Setaria verticillata* | 1994 | Santiago | 15.016667 | -23.583333 | 623 |
| *Setaria verticillata* | 1994 | Santiago | 15.016667 | -23.583333 | 623 |
| *Setaria verticillata* | 1994 | Santiago | 15.016667 | -23.583333 | 623 |
| *Setaria verticillata* | 1994 | Santiago | 15.033333 | -23.633333 | 805 |
| *Setaria verticillata* | 1994 | Santiago | 15.233333 | -23.733333 | 176 |
| *Setaria verticillata* | 1994 | Santiago | 15.1 | -23.683333 | 394 |
| *Setaria verticillata* | 1994 | Santiago | 15.233333 | -23.733333 | 176 |
| *Setaria verticillata* | 1994 | Santiago | 15.083333 | -23.65 | 625 |
| *Setaria verticillata* | 1994 | Santiago | 15.083333 | -23.65 | 625 |
| *Setaria verticillata* | 1994 | Santiago | 15.083333 | -23.65 | 625 |
| *Setaria verticillata* | 1994 | Santiago | 15.05 | -23.6 | 347 |
| *Setaria verticillata* | 1994 | Santiago | 15.05 | -23.6 | 347 |
| *Setaria verticillata* | 1994 | Santiago | 15.083333 | -23.65 | 625 |
| *Setaria verticillata* | 1994 | Santiago | 15.083333 | -23.65 | 625 |
| *Setaria verticillata* | 1994 | Santiago | 15.05 | -23.616667 | 434 |
| *Setaria verticillata* | 1992 | Santiago | 14.933333 | -23.566667 | 107 |
| *Setaria verticillata* | 1992 | Santiago | 15.0 | -23.533333 | 287 |
| *Setaria verticillata* | 1992 | Santiago | 15.05 | -23.533333 | 434 |
| *Setaria verticillata* | 1992 | Santiago | 14.916667 | -23.616667 | 23 |
| *Setaria verticillata* | 1992 | Santiago | 15.216667 | -23.716667 | 404 |
| *Setaria verticillata* | 1992 | Santiago | 15.066667 | -23.633333 | 552 |
| *Setaria verticillata* | 1992 | Santiago | 15.066667 | -23.583333 | 216 |
| *Setaria verticillata* | 1992 | Santiago | 15.033333 | -23.633333 | 805 |
| *Setaria verticillata* | 1992 | Santiago | 15.033333 | -23.633333 | 805 |
| *Setaria verticillata* | 1992 | Santiago | 15.1 | -23.55 | 94 |
| *Setaria verticillata* | 1992 | Santiago | 15.1 | -23.55 | 94 |
| *Setaria verticillata* | 1992 | Santiago | 15.1 | -23.55 | 94 |
| *Setaria verticillata* | 1992 | Santiago | 15.066667 | -23.566667 | 168 |
| *Setaria verticillata* | 1992 | Santiago | 15.066667 | -23.566667 | 168 |
| *Setaria verticillata* | 1992 | Santiago | 15.05 | -23.633333 | 1037 |
| *Setaria verticillata* | 1976 | São Vicente | 16.8717 | -24.9331 | 700 |
| *Setaria verticillata* | 1964 | Maio | 15.1806 | -23.2131 | 24 |
| *Sorghum arundinaceum* | 1956 | Fogo | 14.983083 | -24.434406 | 980 |
| *Sorghum bicolor* | 1956 | Brava | 14.864712 | -24.721893 | 303 |
| *Sorghum bicolor* | 1934 | Fogo | 14.941866 | -24.459109 | 650 |
| *Sorghum bicolor* | 1934 | Fogo | 14.941995 | -24.458993 | 655 |
| *Sorghum bicolor* | 1955 | Santiago | 15.293155 | -23.713060 | 177 |
| *Sorghum bicolor* | 1983 | Santiago | 15.107299 | -23.722003 | 344 |
| *Sorghum bicolor* | 1983 | Santiago | 15.165459 | -23.583738 | 30 |
| *Sorghum bicolor* | 1908 | Santiago | 14.951326 | -23.556185 | 143 |
| *Sorghum bicolor* | 1934 | Santo Antão | 17.126718 | -25.080914 | 1200 |
| *Sorghum halepense* | 1983 | Brava | 14.875183 | -24.724893 | 352 |
| *Sorghum halepense* | 1955 | Santiago | 15.249647 | -23.699168 | 283 |
| *Sorghum halepense* | 1955 | Santiago | 15.292924 | -23.713386 | 180 |
| *Sorghum halepense* | 1955 | Santiago | 15.009026 | -23.519765 | 230 |
| *Sorghum halepense* | 1980 | Santiago | 15.107072 | -23.670148 | 527 |
| *Sorghum halepense* | 1981 | Santiago | 15.117720 | -23.604396 | 300 |
| *Sorghum halepense* | 1981 | Santiago | 15.146402 | -23.563587 | 15 |
| *Sorghum halepense* | 1992 | Santiago | 15.215507 | -23.712181 | 465 |
| *Sorghum halepense* | 1994 | Santiago | 15.109505 | -23.677048 | 545 |
| *Sorghum halepense* | 1994 | Santiago | 15.017325 | -23.569132 | 430 |
| *Sorghum halepense* | 1998 | Santiago | 15.027220 | -23.579779 | 600 |
| *Sorghum halepense* | 1994 | Santiago | 15.089424 | -23.672419 | 540 |
| *Sorghum halepense* | 1994 | Santiago | 15.064944 | -23.678002 | 680 |
| *Sorghum halepense* | 2006 | São Nicolau | 16.628644 | -24.327783 | 616 |
| *Sorghum halepense* | 2006 | São Nicolau | 16.628546 | -24.327784 | 616 |
| *Sorghum halepense* | 1994 | Santiago | 15.016667 | -23.6 | 768 |
| *Sorghum halepense* | 1994 | Santiago | 15.016667 | -23.6 | 768 |
| *Sorghum halepense* | 1994 | Santiago | 15.016667 | -23.583333 | 623 |
| *Sorghum halepense* | 1994 | Santiago | 15.016667 | -23.583333 | 623 |
| *Sorghum halepense* | 1994 | Santiago | 15.233333 | -23.733333 | 176 |
| *Sorghum halepense* | 1994 | Santiago | 15.1 | -23.683333 | 394 |
| *Sorghum halepense* | 1994 | Santiago | 15.233333 | -23.733333 | 176 |
| *Sorghum halepense* | 1994 | Santiago | 15.216667 | -23.716667 | 404 |
| *Sorghum halepense* | 1992 | Santiago | 15.216667 | -23.716667 | 404 |
| *Sorghum halepense* | 1972 | Santiago | 15.0244 | -23.5833 | 500 |

**Supplementary Table 3.** Priority score of the CWR Poaceae occurring in Cabo Verde.

| **Taxon** | **Priority score** |
| --- | --- |
| *Digitaria horizontalis* | 15 |
| *Avena barbata* | 16 |
| *Digitaria nuda* | 17 |
| *Setaria verticillata* | 17 |
| *Digitaria ciliaris* | 18 |
| *Setaria pumila* | 18 |
| *Avena sativa* | 18 |
| *Eragrostis cilianensis* | 18 |
| *Eragrostis ciliaris* | 18 |
| *Echinochloa crus-galli* | 19 |
| *Eleusine indica* | 19 |
| *Panicum laetum* | 19 |
| *Cenchrus pedicellatus* | 19 |
| *Echinochloa colona* | 20 |
| *Digitaria eriantha* | 20 |
| *Digitaria nodosa* | 20 |
| *Digitaria sanguinalis* | 20 |
| *Hordeum vulgare* | 21 |
| *Avena fatua* | 21 |
| *Sorghum halepense* | 21 |
| *Imperata cylindrica* | 21 |
| *Eragrostis pilosa* | 22 |
| *Cenchrus polystachios ssp. atrichus* | 23 |
| *Paspalum scrobiculatum* | 24 |
| *Sorghum bicolor* | 24 |
| *Sorghum arundinaceum* | 25 |

**Supplementary Table 4.** Number of priority CWR Poaceae species per island and their priority categories.

| **Island** | **Priority categories** | | | **Total** |
| --- | --- | --- | --- | --- |
|  | **Low** | **Medium** | **High** |  |
| **Santo Antão** | 9 | 9 | 3 | 21 |
| **São Vicente** | 5 | 3 | 0 | 8 |
| **Santa Luzia** | 1 | 0 | 0 | 1 |
| **São Nicolau** | 5 | 4 | 0 | 9 |
| **Sal** | 3 | 0 | 0 | 3 |
| **Boavista** | 3 | 1 | 1 | 5 |
| **Maio** | 7 | 2 | 0 | 9 |
| **Santiago** | 8 | 9 | 4 | 21 |
| **Fogo** | 7 | 6 | 3 | 16 |
| **Brava** | 6 | 2 | 2 | 10 |

**Supplementary Table 5.** Details on the Poaceae crops studied, namely their common and scientific names, score of crop importance concerning to the food supply and agricultural production metrics of the crops, crop native distribution, number of CWRs occurring in Cabo e and the mean priority score of the CWRs.

| **Crop common name** | **Crop scientific name** | **Crop importance score** | **Crop native distribution** | **Number of CWR in Cabo Verde** | **Priority score of CWR (mean)** |
| --- | --- | --- | --- | --- | --- |
| Barley | *Hordeum vulgare* | 0.504 | Africa excl. West | 1 | 21.0 |
| Barnyard millet | *Echinochloa colona, Echinochloa crus-galli* | 0.304 | Only out of Africa | 2 | 19.5 |
| Finger millet | *Eleusine coracana, Eleusine coracana subsp. coracana* | 0.189 | Africa incl. West | 1 | 19.0 |
| Fonio / White fonio | *Digitaria exilis, Digitaria iburua* | 0.304 | Africa incl. West | 6 | 18.3 |
| Foxtail millet / Broom millet | *Setaria italica* | 0.383 | Africa excl. West | 2 | 17.5 |
| Indian Barnyard millet / White millet / Siberian millet | *Echinochloa frumentacea* | 0.203 | Only out of Africa | 2 | 18.5 |
| Japanese Barnyard millet | *Echinochloa esculenta* | 0.203 | Only out of Africa | 1 | 19.0 |
| Kodo millet | *Paspalum scrobiculatum* | 0.210 | Africa incl. West | 1 | 24.0 |
| Oats | *Avena sativa* | 0.537 | Only out of Africa | 3 | 18.3 |
| Pearl millet | *Cenchrus americanus (=Pennisetum glaucum )* | 0.449 | Africa incl. West | 2 | 21.0 |
| Proso millet | *Panicum miliaceum* | 0.272 | Only out of Africa | 1 | 19.0 |
| Sorghum | *Sorghum bicolor* | 0.469 | Africa incl. West | 3 | 23.3 |
| Sugarcane | *Saccharum officinarum* | 0.502 | Only out of Africa | 2 | 20.5 |
| Teff (millet) | *Eragrostis tef* | 0.254 | Africa excl. West | 3 | 19.3 |
